# Biomarker Responses, Gene Expression Alterations, and Histological Changes in Zebrafish (*Danio rerio*) After *In Vivo* Exposure to Polychlorinated Diphenyl Ethers

**DOI:** 10.3389/fphys.2022.907906

**Published:** 2022-06-03

**Authors:** Chunmeng Ye, Wenli Xiong, Shuaishuai Shi, Jiaqi Shi, Wenhui Yang, Xuesheng Zhang

**Affiliations:** ^1^ School of Resources and Environmental Engineering, Anhui University, Hefei, China; ^2^ Laboratory of Wetland Protection and Ecological Restoration, Anhui University, Hefei, China; ^3^ Nanjing Institute of Environmental Sciences of the Ministry of Ecology and Environment, Nanjing, China

**Keywords:** polychlorinated diphenyl ethers, oxidative stress, histological changes, integrated biomarker response, endocrine disrupting effects

## Abstract

Polychlorinated diphenyl ethers (PCDEs) have been detected in various aquatic matrices, which pose potential threats to aquatic ecosystem security. In this work, both micro and macro analysis methods were used to assess the toxicity of PCDEs to zebrafish. Results indicated that after *in vivo* PCDE exposure, the oxidative stress and related gene of *Danio rerio* were significantly changed. The higher concentration or longer exposure time could cause more severe oxidative stress in zebrafish tissues. Compared with among the five tested compounds, more obvious changes in the level of oxidative biomarkers of lower chlorinated PCDEs’ (4-mono-CDE and 4,4′-di-CDE) exposure groups were observed. The integrated biomarker response analysis and gene expression results also indicate a similar trend. Histopathological observation suggested that 4,4′-di-CDE could render liver nuclei enlargement and necrosis, hepatocyte vacuolation, and the development inhibition of ovarian cells. Transmission electron microscope photos showed that 4,4′-di-CDE caused organelle damage in the liver and ovary, including the rupture of the endoplasmic reticulum, swelling of mitochondria, and condensation of chromatin in the liver and mitochondria disappeared significantly in the ovary. The degree of damage is enhanced with the increasing exposure doses. In addition, PCDEs also significantly altered vitellogenin content and related gene (*vtg1*) expression, suggesting that PCDEs may be estrogen endocrine disruptors. Overall, these results provided some valuable toxicological data of PCDEs on aquatic species.

## 1 Introduction

Polychlorinated diphenyl ethers (PCDEs) are a class of emerging organic contaminants that have 209 homologs, whose structures resemble polychlorinated biphenyls (PCBs) and dioxins (Domingo, 2006; Zeng et al., 2007; Li et al., 2012). PCDEs have been widely used as insulating oil, flame retardants, lubricants, and plasticizer in the 20th century (Koistinen, 2000; Dann and Hontela, 2011). PCDEs in the environment mainly originate from residuals in the production of chlorophenol (or sodium chlorophenol), waste incineration, and especially the incomplete combustion containing chlorine substances (Nilsson and Renberg, 1974; Kerkvliet et al., 1985; Kurz and Ballschmiter, 1995).

PCDE congeners have been prevalently detected in various environmental matrices (Supplementary Table S1), especially in aquatic environmental samples (Bocio et al., 2004; Persson et al., 2007). For instance, fifteen types of PCDEs were detected in surface water, sediment, and suspended particulate matter (SPM) with concentrations reaching 0.351–2.021 ng/L, 0.279–2.474 ng/g d.w., and 0.331–2.013 ng/g d.w. in the Chaohu Lake (Zhang et al., 2018). Seven types of PCDEs were detected in the sediment of the Great Lakes (ND-5.54 ng/g d.w.) (Li et al., 2018). Fifty types of PCDEs were detected in the sediment of the Kymijoki river (138–561 ng/g d.w.) (Koistinen et al., 1995a). In addition, PCDEs were also detected in biological samples, such as fish samples (2.1–21 ng/g l.w.) (Koistinen et al., 1995b; Sinkkonen and Paasivirta, 2000).

Considering the widespread distribution and high bioaccumulation potential of PCDEs in aquatic species, their adverse impacts on aquatic organisms have been partly investigated. Previous research demonstrated that PCDEs can induce hepatotoxicity and developmental, reproductive, and thyroid toxicity in different model organisms, but the toxicity research on aquatic organisms is still limited (Iverson et al., 1987; Rosiak et al., 1997; Qin et al., 2014; Zhang et al., 2014; Fang et al., 2018). Chui et al. (1990) reported that the acute toxicity (96 h-*LC*_50_) values of 4-mono-CDE and 2,4-di-CDE were correspondingly 0.73 and 0.66 mg/L for trout, and these two substances have the highest enrichment rates in their blood and liver than that in the adipose tissue and muscle. 4,4′-di-CDE was considered to affect the morphological development process of zebrafish embryos, inhibit the growth of green algae, and reduce the content of algal cytochrome (Qin et al., 2014; Fang et al., 2018). Cheng et al. (2019) investigated the oxidative stress level caused by 3,4,4′-tri-CDE, 2-MeO-3′,4,4′-tri-CDE, and 2-OH-3′,4,4′-tri-CDE to crucian carp, which revealed the liver stress response from a macro perspective. However, the specific situation and mechanism of liver damage have not been deeply explored. Given the lack of research on the oxidative damage and mechanism caused by different PCDEs to aquatic organisms, it is necessary to carry out research to explore related aspects.

When organisms are exposed to certain environmental pollutants, they may stimulate the high production of reactive oxygen species (ROS), which leads to cell failure and the release of malondialdehyde (MDA) (Livingstone, 2003). To prevent oxidative stress, organisms and cells are equipped with endogenous defense systems, including enzymatic antioxidants [e.g., superoxide dismutase (SOD), catalase (CAT), and glutathione peroxidase (GPx) activity] and nonenzymatic antioxidants [e.g., glutathione (GSH)]. These biomarkers associated with ROS have been widely used to evaluate and compare the adverse effects of environmental pollutants’ exposure to water on aqueous species (Burgos-Aceves et al., 2018). Furthermore, the production of ROS is also responsible for inducing the expression of many genes, while the transcriptomic responses are rapid (Lettieri, 2006; Chen et al., 2011; Oggier et al., 2011). The induction of vitellogenin (VTG), which can be used to screen estrogen endocrine disruptors, has been proven to be effective and reliable (Li et al., 2021). VTG is a yolk precursor protein, which is synthesized in the liver and responded to estrogen signals by binding to nuclear estrogen receptor (ER) in hepatocytes (Fernández-González et al., 2020). It has been found that adult male and juvenile zebrafish can synthesize yolk proteinogen in response to exogenous estrogen induction, and the transcription of yolk proteinogen gene mRNA is significantly upregulated (Andersen et al., 2003; Petersen and Tollefsen, 2012; Zhu et al., 2017). Due to the high lipophilicity and stability of PCDEs, attention has been mainly focused on ecological risks and adverse effects of PCDEs. However, the basic data on the toxic effects of PCDEs are relatively scarce.

Considering the structural characteristics of PCDEs, we speculate that they may have toxic effects similar to PCBs and PBDEs on aquatic organisms (such as zebrafish), for example, inducing oxidative stress, tissue damage, and endocrine interference. However, there is no exact report on this work at present. Hence, the aims of the current study include 1) exploring changes in five typical antioxidant biomarkers SOD, CAT, GPx, and GSH as well as the degree of lipid peroxidation indicator (MDA) and gene expression levels; 2) studying the toxic effects of PCDE exposure on the VTG concentration change and *vtg1* gene expression level; and 3) observing the histopathological changes of the liver and ovarian tissues to determine the potential organ lesions induced by PCDE exposure. In addition, the toxicity order of these compounds on aquatic species was discussed based on the integrated biomarker response (IBR) index. This work could provide valuable toxicological data about PCDEs on aquatic species, which can facilitate to evaluation of the threat of PCDEs to the ecological environment and human health.

## 2 Materials and Methods

### 2.1 Chemicals and Reagents

**FIGURE 1:**
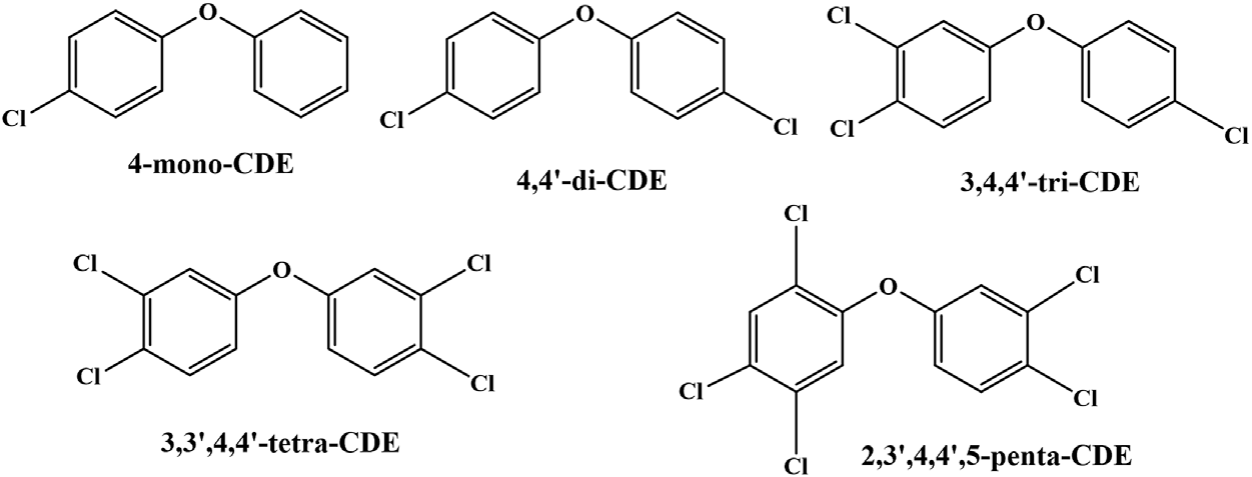
The structures of five tested PCDEs.

Different PCDE congeners (4-mono-CDE, 4,4′-di-CDE, 3,4,4′-tri-CDE, 3,3′,4,4′-tetra-CDE, and 2,3′,4,4′,5-penta-CDE) were synthesized according to our previous study, of which the purity (> 99.0%) met our previous experimental requirements (Zhang et al., 2015; Yang et al., 2022). Their specific structures are shown in Figure 1. The kits for vitellogenin and oxidative stress biomarkers were supplied by Shanghai Enzyme-linked Biotechnology Co., Ltd. (Shanghai, China) and Nanjing Jiancheng Bioengineering Institute (Nanjing, China), respectively. The other reagents and materials regarding gene detection are listed in the Supplementary Material.

### 2.2 Zebrafish Domestication

Wild-type zebrafish (AB strain, 6-month-old) were purchased from the Institute of Hydrobiology of the Chinese Academy of Sciences (Wuhan, China) and placed in an aquarium with a filtration and circulation system (volume: 50 cm × 31 cm × 49 cm). The light was artificially controlled for 14 h per day, and the temperature was controlled at 28 ± 1°C. After feeding (8 p.m. and 5 a.m.), one-third of the water in the aquarium was replaced with dechlorinated tap water, and the tank was cleaned periodically during the domestication period. The quality of the incubation water was as follows: pH, 7.5 ± 0.1; dissolved oxygen, 7 ± 0.5 mg/L; conductivity, 521 ± 9.6 μs/cm; hardness, 130 ± 6.4 mg CaCO_3_/L. No food was provided for zebrafish 24 h before dissection and formal experiment to eliminate the effect of feeding.

### 2.3 Exposure Experiment Design

The current study mainly focused on the subacute toxicity of PCDEs on zebrafish, hence the 14 d was selected as the sampling time point according to the basis of guideline 204 of OECD (2004). Meanwhile, since we try to investigate the time-dependent effects of PCDEs on zebrafish, 3 d, 7 d, and 11 d were also selected. Based on previous toxicity experiments and environmental levels of PCDEs, the exposure doses of PCDEs were 0, 1, 10, and 50 μg/L, respectively (Li et al., 2012; Li et al., 2016; Qin et al., 2015; Yang et al., 2022). The stock solutions of PCDEs were prepared in DMSO and stored at room temperature in the dark. To detect biomarker level, the exposure experiment of five PCDEs was conducted in five batches. In each batch, male zebrafish (900 numbers) were randomly divided into five experimental groups such as the water and solvent group [DMSO (0.4%, v/v)] and three PCDE exposure groups at concentrations of 1, 10, and 50 μg/L. Each group was maintained in three replicates, and each replicate contains 60 fish in a 40 L test solution. In addition, male and female zebrafish (300 numbers) need to be exposed to 4,4′-di-CDE for 14 d and then used for section observation and gene analysis. During the experiment, the water in the original tank was replaced every two days with freshwater containing PCDE solution to ensure a constant concentration of the compounds in the tank. The concentrations of five test compounds were monitored during the whole exposure period. The analytical method for PCDEs extracted from the exposure matrix is also described in the Supplementary Material. PCDEs were analyzed using GC-MS (Trace DSQ II, Thermo Scientific, United States). Compared with the theoretical concentration, the exposure concentration change was within 15%.

### 2.4 Sample Preparation

All the experiments were performed following the laboratory animal welfare of China and were approved by the animal ethics committee of Anhui University (No. 201916; effective date, 15 March 2019). Ten male zebrafish were randomly selected and dissected for livers on the ice after 3, 7, 11, and 14 days. On the 14^th^ day, except for detecting antioxidant biomarkers, male zebrafish livers (10 numbers) and blood (10 numbers) were also used to detect VTG concentration. These liver samples needed to be rinsed (0.86% NaCl), homogenized with NaCl (Ultra Turrax, IKA, Germany), and centrifuged at 8,000 r/min for 10 min at 4°C (Sorvall ST 8R, Thermo, United States) for enzyme-linked immunosorbent assay (ELISA) kits and enzyme activity test according to the manufacturer’s instructions and previous study, respectively. Blood samples were centrifuged (3,500 rpm, 10 min, 4°C) and then the supernatants were diluted (1: 9, v/v) in 0.9% saline. Fifty microliter solutions were used to determine the VTG content.

After 4,4′-di-CDE exposure, the male liver (10 numbers) for gene analysis was preserved in TRIzol reagent for transcriptional expression analysis. The male zebrafish liver and female ovary for section observation were fixed with tissue fixative and 2.5% glutaraldehyde.

### 2.5 Histopathological Examinations

Liver and ovary samples were initially put in tissue fixative. The fixed tissue samples were dehydrated in a series of graded ethanol, embedded in non-solidified paraffin, and cooled at −20°C. Then, they were sectioned at 4 μm thickness and stained with hematoxylin and eosin (H&E) for histopathological analysis (Pearse, 1968; Roberts, 1978; Humason, 1979). The sections were examined and photographed using a light microscope (Olympus BH-2).

### 2.6 Transmission Electron Microscopy Observation

The two test livers and ovaries were separated from the fish and then continued to be fixed with 2.5% glutaraldehyde overnight at 4°C and rinsed with 0.1 M phosphate buffer (pH 7.4). When finished, tissue blocks were fixed in 1% osmic acid that is diluted by 0.1 M phosphate buffer for 2 h, then rinsed and dehydrated for 15 min with ethanol. After embedding in epoxy resin and drying at 55 °C for 48 h, specimens were cut into ultrathin sections (80 nm) by using an ultramicrotome and then stained with lead citrate and uranyl acetate to examine in TEM (JEOL, Tokyo, Japan) at the Sophisticated Analytical Instrument Facility (SAIF).

### 2.7 Biochemical Analysis

SOD, CAT, GPx, GSH, and MDA activity levels were determined according to Flohé and Oyying (1984a), Goth (1991), Flohé and Gunzler (1984b), Diamantino et al. (2001), Claiborne (1985), and Devasagayam (1986). Results of these parameters were expressed as U T-SOD/mg protein, U CAT/mg protein, U GSH-Px/mg protein, mg GSH/protein, and nmol MDA/mg protein, respectively. The protein content and enzyme activity of this experimental sample were tested according to our previous research methods (Yang et al., 2022). Detailed descriptions can be found in the Supplementary Material.

### 2.8 Integrated Biomarker Response

The IBR index was used to integrate all results from different biomarkers and understand general responses. According to Beliaeff and Burgeot’s methods (2002), the IBR value is calculated by summing up triangular star plot areas calculated for each pair of neighboring data. Normalized data can be seen in the Supplementary Material.

### 2.9 Quantitative Real-Time PCR Experiments (qRT-PCR)

TRIzol method was applied to extract the total RNA from zebrafish liver tissues. The integrity of total RNA was detected by 1.5% agarose gel electrophoresis, which concentration and OD_260_/OD_280_ (1.8–2.2) value meeting the quality requirements would be selected for subsequent tests. The relevant steps are listed in the Supplementary Material. Complementary DNA (cDNA) was synthesized from 2 μg total RNA using the PrimeScript® RT Master Mix (TakaRa, Dalian, China) reverse transcription kit. Quantitative real-time PCR (qRT-PCR) amplification was carried out using CFX96™ Real-Time PCR detection system (Bio-Rad, United States). SYBR Green I (Bio-Rad, United States) and Prime Script RT Master Mix kit (DRR036A) methods (TaKaRa) were also carried, with β-actin gene as the endogenous control. The relative expression stability of the reference genes was calculated using geNorm and the data showed expression stability values of 0.38 for *β*-actin. Relative expression data were geometrically normalized to *β*-actin (Perrichon et al., 2016). Primer sequences were synthesized by Mingke Biotechnology (Hangzhou) Co., Ltd., and have been tabulated in Supplementary Table S2. qRT-PCR was performed with three biological replicates and three technical replicates. Statistical analyses were carried out on the group mean values using a random reallocation test. The PCR conditions were as follows: 40 cycles of amplification at 95 °C for 10 s, annealing at 57 °C for 15 s, and extension at 72 °C for 30 s. Standard curves were constructed for each gene using serial dilutions of stock cDNA. The amplification efficiencies of all genes were approximately equal and ranged from 94.01% to 100.49%. Fold changes were calculated according to the 2^−ΔΔCt^ method (Livak and Schmittgen, 2001).

### 2.10 Statistical Analysis and Data Processing

Statistical analyses were processed by SPSS 25.0 (Chicago, United States) and GraphPad Prism 5.0 (San Diego, United States). The difference of controls (negative and solvent) was carried out using a Tukey (*p <* 0.05). Prior to one-way ANOVA analysis, Kolmogorov–Smirnov and Levene tests were used to check the normality distribution and homogeneity of variances in experimental data. A one-way analysis of variance (ANOVA) followed by Tukey’s multiple comparison test was used to analyze the significant difference (*p* < 0.01 and *p* < 0.05) between each exposure group and the control group.

## 3 Results and Discussion

### 3.1 Effects on Oxidative Stress Biomarkers

#### 3.1.1 Redox Enzyme Activities

**FIGURE 2:**
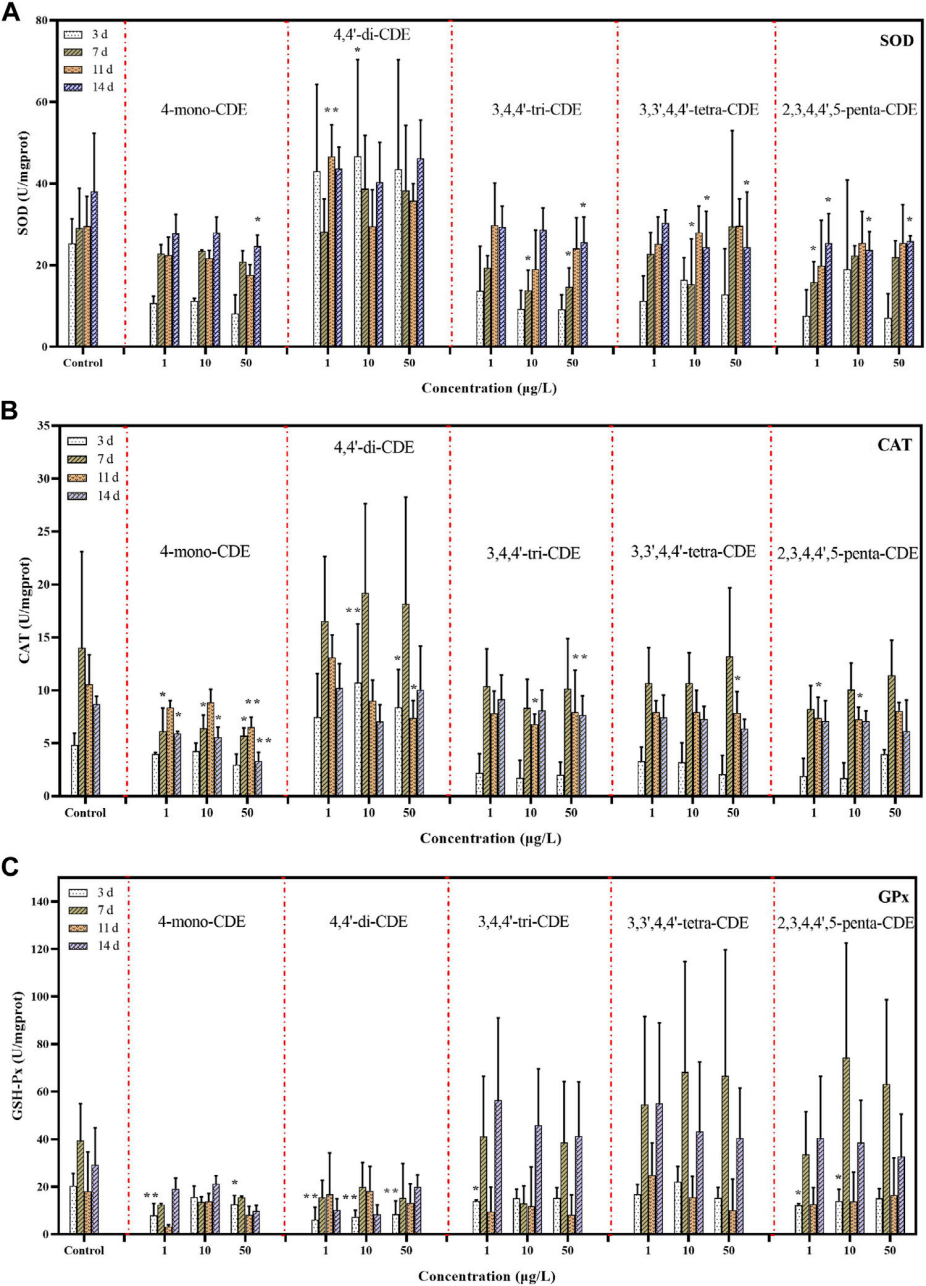
The effects of PCDEs on the antioxidant defense system. SOD **(A)**, CAT **(B)**, and GPx **(C)** in zebrafish liver.

The activities of SOD, CAT, and GPx in the liver of male zebrafish exposed to PCDEs are depicted in Figure 2. The concentration of PCDEs and exposure time have a great impact on the stress level of zebrafish. The higher concentration or longer exposure time would cause an obvious change in the oxidative stress level. Specifically, except for 4,4′-di-CDE, the other mid-high concentration of PCDE exposure groups inhibited the level of SOD and CAT on zebrafish livers, and the same inhibition was also manifested in the extension of exposure time.

Compared to the control group, except for 4,4′-di-CDE, the SOD activity of most poisoned groups decreased to varying degrees in zebrafish during the exposure period (Figure 2A). After exposure to different concentrations of 4,4′-di-CDE, relative to the control group, the SOD activity in zebrafish liver increased to varying degrees. Exposure to the 7^th^ day and 14^th^ day, except for 4,4′-di-CDE, the SOD activity showed inhibition (*p* < 0.05) in the 10 and 50 μg/L dose groups of PCDEs. In addition, SOD activity was increased with the extension of exposure time in the experimental group, which indicated the longer exposure time to PCDEs, the more severe damage to the organism. We also observed that SOD activity was more easily inhibited significantly in medium–higher concentration treating groups compared to the control group. These results were consistent with the research that benzotriazole ultraviolet stabilizer-328 in the later stages of exposure can cause SOD levels to decrease in zebrafish (Hemalatha et al., 2020). However, some research was contrary to the current study, such as the activity of SOD was increased in a concentration-dependent manner after exposure to di-n-butyl phthalate and diethyl phthalate in zebrafish embryos (Xu et al., 2013). This difference might be attributed to the varying substance concentrations, different treatment durations, and alterations between different species.

Similar to the changes in SOD activity, CAT activity in fish liver treated with 4,4′-di-CDE also presented an increasing trend, especially for the high concentration groups (Figure 2B). No statistical difference in CAT activity between the control and treatment groups when the exposure time was 3 days. The minor variations of CAT activity were observed on the 7^th^ day and 14^th^ day, while a significant decrease (*p* < 0.05) occurred only in the treatments by 4-mono-CDE. Conspicuous inhibitions were detected for every PCDE on the 11th day (*p* < 0.05 or *p* < 0.01). In addition to that, the trend of CAT activity was suppressed in the exposure later period, which implied the zebrafish liver-produced redundant ROS to attack the cells, resulting in the synthesis of SOD and CAT might be restricted.

The hepatic antioxidant system can degrade ROS that is produced by intracellular macromolecules (Morales et al., 2004; Huang et al., 2007). As the first defensive line of the antioxidant defense system, SOD safeguards the cells against free radical-induced oxidative damage by catalyzing the conversion of superoxide anion (O^2−^) to molecular oxygen (O_2_) and hydrogen peroxide (H_2_O_2_), while CAT is responsible for removing the H_2_O_2_ from the system by converting it to water (H_2_O) and oxygen (O_2_) (Zhu et al., 2008). In our study, the SOD and CAT activities increased significantly after exposure to 4,4′-di-CDE for 3 days, indicating that the antioxidant mechanism may be self-protective cleaning of ROS (Sun et al., 2006). However, for most treatment groups, the SOD activity was inhibited and the CAT activity also showed a downward trend with the increase in exposure time. This phenomenon illustrated that more PCDEs may accumulate in organisms, resulting in the excessive production of reactive oxygen species (ROS). Those redundant ROS can attack the cells, and the synthesis of SOD and CAT might be restricted, leading to significant decreases in their activities. For the 4-mono-CDE-treated group, the activities of SOD and CAT exhibited a similar pattern during the whole exposure process, showing a downward trend, indicating that this group may produce the most serious intensity of oxidative stress. These data indicated that this group may produce the most serious intensity of oxidative stress. SOD and CAT activities were more sensitive to the low-chlorinated congener-treated groups (i.e., 4-mon-CDE and 4,4′-di-CDE).

For GPx, 4-mono-CDE and 4,4′-di-CDE exposure groups showed inhibition of oxidative stress levels, while the rest showed promotion but not significant (Figure 2C). Otherwise, it was noted that GPx activity was remarkably inhibited (*p* < 0.05 or *p* < 0.01) in the low concentration groups on the 3^rd^ day. As the second defense enzyme against oxidative stress, GPx can not only terminate the diffusion of free radical chain reaction but also participates in the decomposition process of hydrogen peroxide and lipid peroxide (Kavitha et al., 2011). For most experimental substances, the GPx activity was not very obvious, which may be related to the sequence of antioxidant enzymes for protection against oxidative damage (Pandey et al., 2003). It is worth noting that GPx activity showed significant inhibition in the low-chlorinated treating groups, suggesting that the hydroperoxide products may overwhelm the antioxidant defense and thus impair GPx synthesis. We can learn from the aforementioned SOD, CAT, and GPx activity test results that low-chlorinated PCDEs may cause damage to the antioxidant system of aquatic organisms even if they are exposed to water at low concentrations.

#### 3.1.2 GSH Levels

**FIGURE 3:**
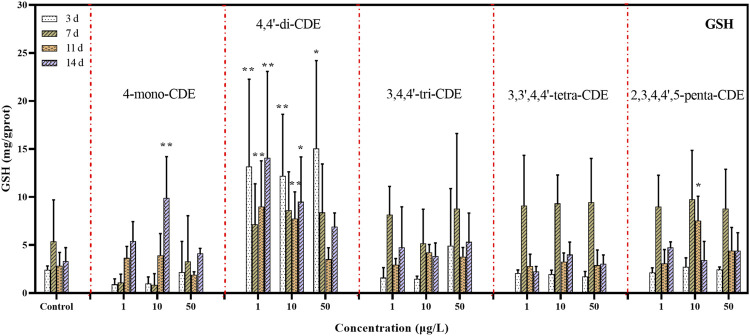
The effect of PCDEs on the GSH levels in zebrafish liver.

GSH is an oxyradical scavenger, that not only plays a crucial role in scavenging free radicals and detoxifying electrophilic compounds but also can interact directly with some reactive oxygen species for cell detoxification (Patrick, 2002; Huang et al., 2010). In our study, as seen in Figure 3, the GSH level increased significantly in the 4,4′-di-CDE experimental group compared with the control group, while the other four homolog test groups showed no significant difference, except 4-mono-CDE (10 μg/L) and 2,3′,4,4′,5-penta-CDE (10 μg/L). This result indicated that GSH may be the most sensitive to 4,4′-di-CDE. In the medium-dose of 4-mono-CDE and 2,3′,4,4′,5-penta-CDE groups, GSH levels were significantly induced in the later stages of the exposure experiment, suggesting that the synthesis of GSH was activated due to the compensatory responses.

In addition, in the 3,4,4′-tri-CDE, 3,3′,4,4′-tetra-CDE, and 2,3′,4,4′,5-penta-CDE exposure groups, the downward trend of GSH levels appeared in the later stage of exposure. This phenomenon indicated that the synthesis rate of GSH may be less than its oxidation rate, and then resulting in the destruction of the intracellular redox state so that oxyradical exceeds the metabolic capacity of the liver.

#### 3.1.3 MDA Contents

**FIGURE 4:**
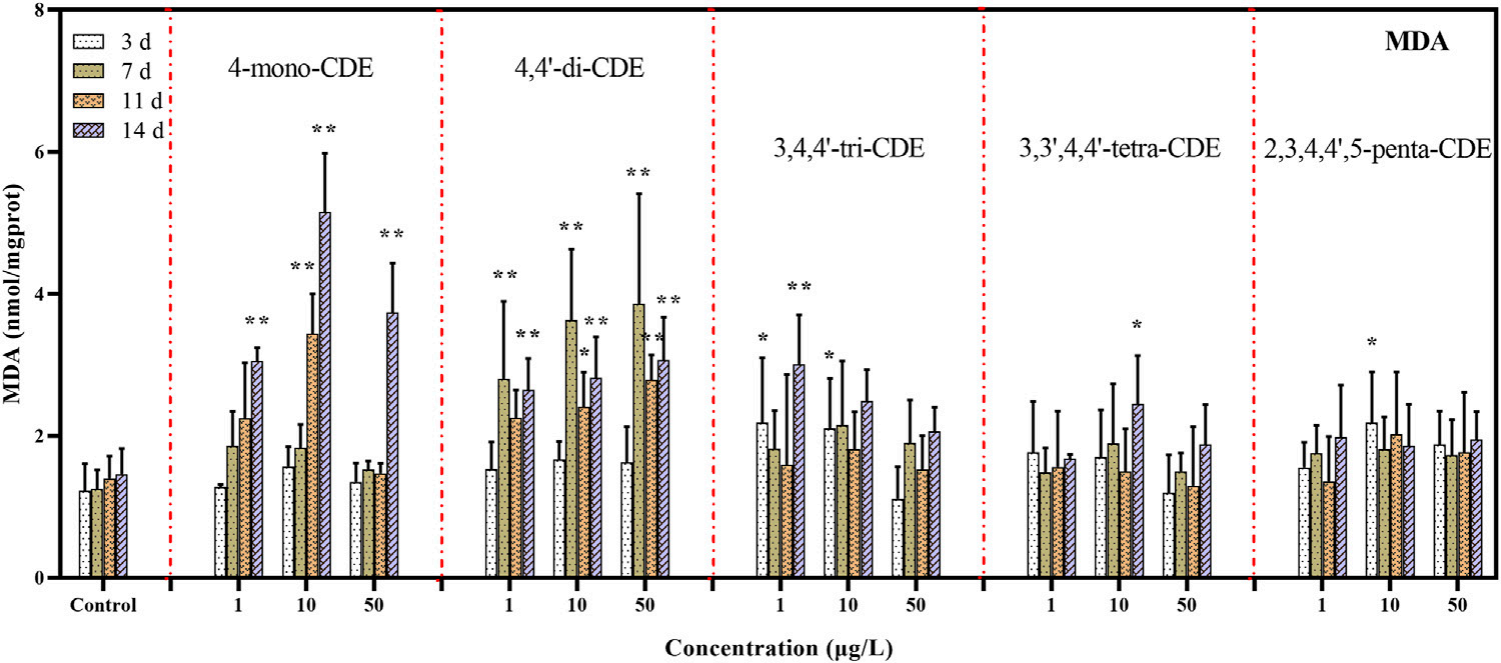
The effect of PCDEs on the MDA levels in zebrafish liver.

With the increasing concentrations of ROS, the unsaturated fatty acids on the cell with membrane surface are degraded to produce lipid peroxide (LPO), which eventually leads to cell failure and the release of malondialdehyde (MDA) (Storey, 1996). The ineffective antioxidant defense system will cause the concentration of MDA to increase, which has a strong destructive effect on the cell membrane and has been considered as the decisive factor for oxidative stress in organisms exposed to various pollutants (Bebianno et al., 2005). In this research (Figure 4), except 3,4,4′-tri-CDE (1 and 10 μg/L) and 2,3′,4,4′,5-penta-CDE (10 μg/L), there was no significant change in other experimental groups on the 3^rd^ day (*p* < 0.05), Only 4,4′-di-CDE experimental groups had a significant change on the 7^th^ day (*p* < 0.01). On the 11^th^ day, the MDA content of 4-mono-CDE (10 μg/L) and 4,4 (10 and 50 μg/L) was significantly higher than that of the control group (*p* < 0.01 or *p* < 0.05). Except for 2,3′,4,4′,5-penta-CDE, the MDA level was highly induced in the other 4 PCDE congeners treated groups (*p* < 0.01 or *p* < 0.05) on the 14^th^ day. For the 4-mono-CDE exposure group, the MDA level increased with exposure time. This phenomenon was similar to the previous study in that the MDA content would be dramatically provoked in the fish liver after prolonged exposure to benzotriazole ultraviolet stabilizer-328 with exposure time (Hemalatha et al., 2020). In the 4,4′-di-CDE exposure group, the MDA content increased significantly on the 3^rd^ day, and then it would fall back, which means that 4,4′-di-CDE may cause great damage to the fish liver and lead to hepatocyte apoptosis. It can be seen that MDA content increased significantly in low-chlorinated PCDE groups (4-mono-CDE and 4,4′-di-CDE), while MDA content did not change significantly in other groups. This result indicated that 4-mono-CDE and 4,4′-di-CDE may cause a more rapid accumulation of ROS to attack the cell membrane and then aggravate the lipid peroxidation. More studies suggested that exposure to 4,4′-di-CDE can lead to significantly enhanced MDA levels in green algae *Scenedesmus obliquus* and exposure to 2-HO-3′,4,4′-tri-CDE showed a more obvious growth trend of MDA content in crucian carp (Fang et al., 2018).

Our previous studies have also suggested that low-chlorinated PCDEs have higher toxicity than middle-chlorinated PCDEs (Fang et al., 2018; Zhang et al., 2018; Yang et al., 2022). Therefore, we can conclude a conclusion that the low-chlorinated PCDEs may more easily induce ROS in fish liver, resulting in great damage to the antioxidation system, attacking the cell membrane, and then exacerbating hepatocyte damages.

### 3.2 Integrated Biomarker Response

The IBR index has proved to be a valuable parameter to evaluate the overall stress of fish liver in different treatment groups (Kim et al., 2010). Normally, lower biomarker scores are indicative of a better health state, while higher scores usually indicate that organisms are in a poorer physiological condition (Yan et al., 2016).

**FIGURE 5:**
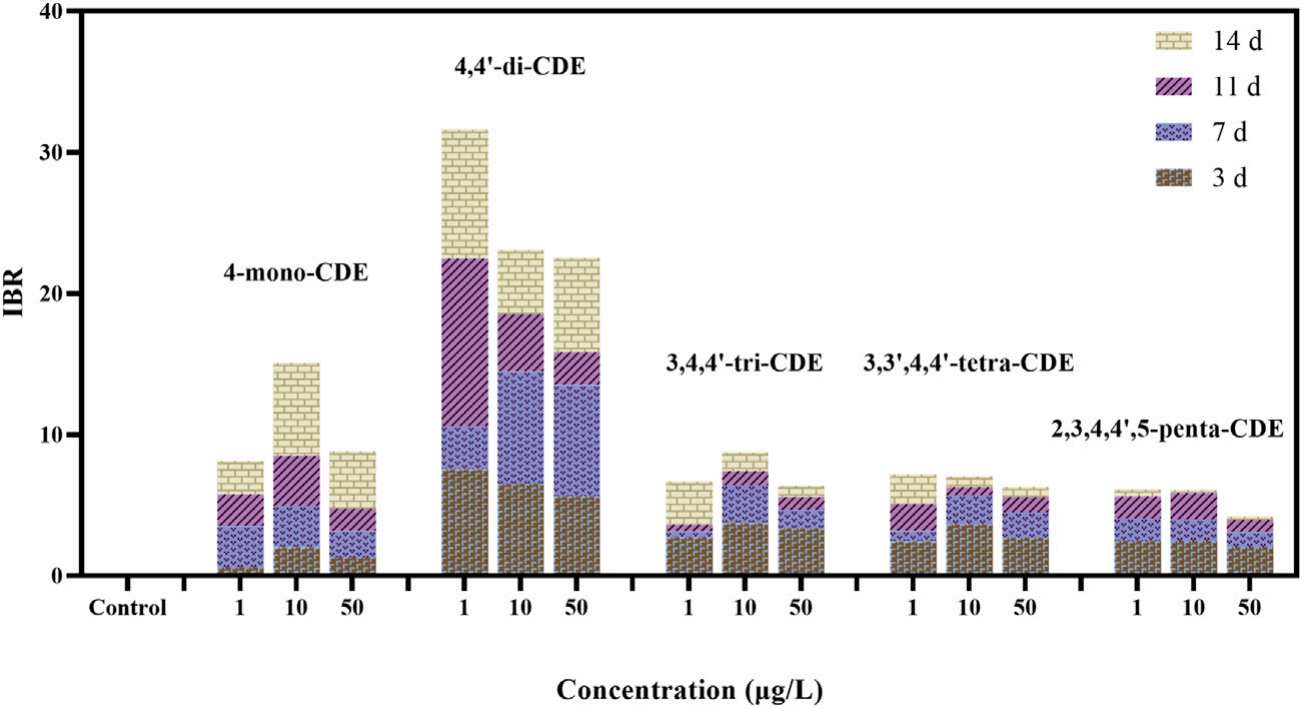
The calculated IBR values of PCDEs for different doses after 14 days of exposure.

In this study, the IBR values are shown in Figure 5 after exposure to 3, 7, 11, and 14 days PCDEs. Their values ranged from 4.2 (2,3′,4,4′,5-penta-CDE, 10 μg/L) to 31.6 (4,4′-di-CDE, 1 μg/L). After exposure for 3, 7, 11, and 14 days, the ranges were from 0.00 (control) to 7.53 (1 μ/L 4,4′-di-CDE), 0.00 (control) to 7.95 (10 μ/L 4,4′-di-CDE), 0.00 (control) to 11.86 (1 μ/L 4,4′-di-CDE), and 0.00 (control) to 9.14 (1 μ/L 4,4′-di-CDE), respectively. It can be seen that 4-mono-CDE and 4,4′-di-CDE (low-chlorinated PCDEs), especially 4,4′-di-CDE, have significantly higher stress on zebrafish than other exposure groups (middle-chlorinated PCDEs), which possibly indicates low-chlorinated PCDEs own higher toxicity. The overall IBR values were higher in the 4,4′-di-CDE exposure group, especially in the early stage and low concentration group, which showed that 4,4′-di-CDE could cause damage to organisms in a short time with low concentration. In other middle chlorination PCDE groups, the IBR value is higher in the mid-high dose and early exposure stage, which exhibited that they can cause serious pressure on organisms in a short time at high concentration. Similar results were also found in *Carassius auratus* exposed to hexabromobenzene and 3,4,4′-tri-CDE (Feng et al., 2014; Fang et al., 2018). Our results implied that the toxicity of low-chlorinated PCDEs was higher than that of mid-chlorinated PCDEs to organisms, which is consistent with the research results of Yang et al. (2022).

### 3.3 Histopathological Changes

**FIGURE 6:**
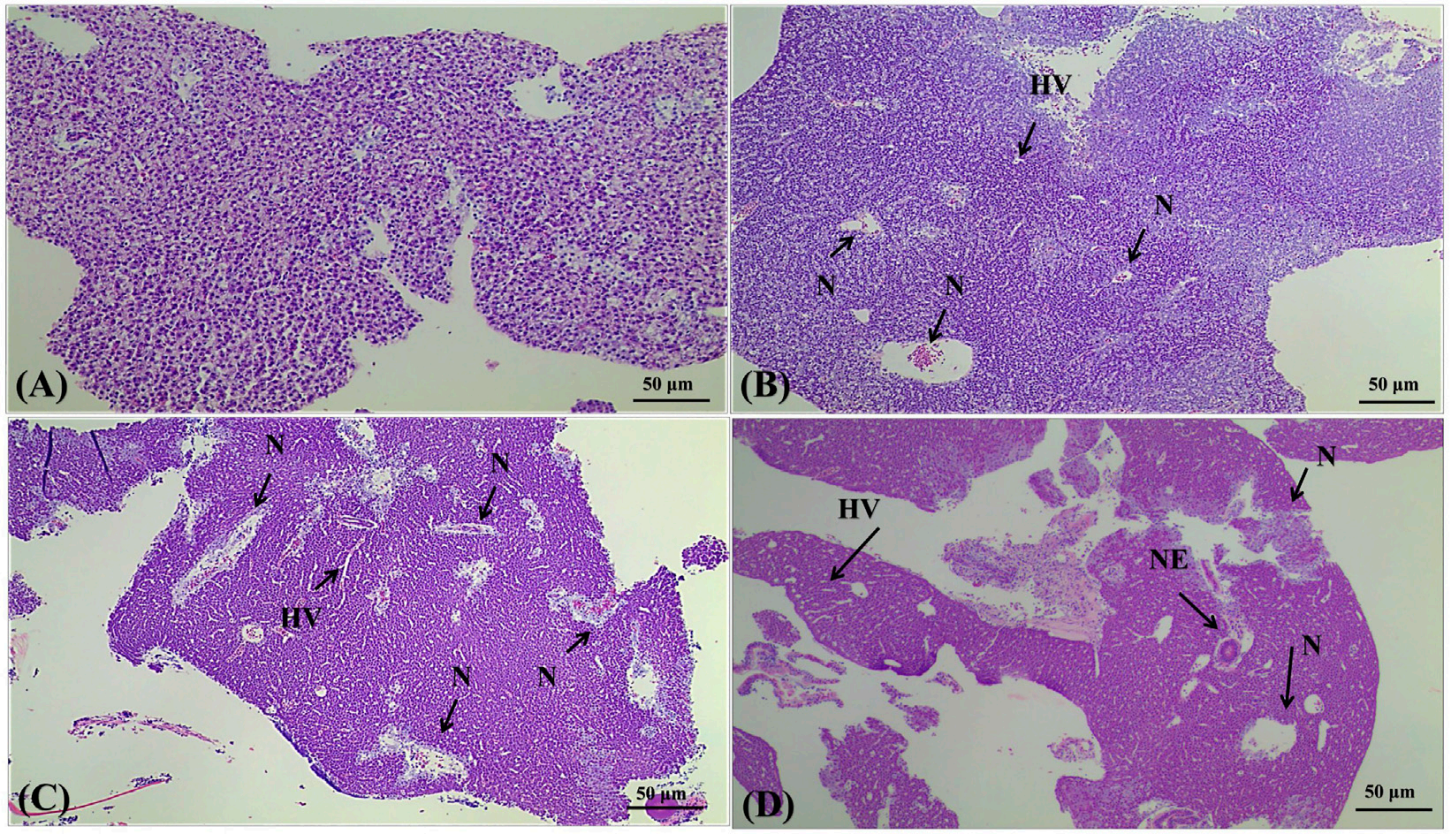
Effects of 4,4′-di-CDE exposure on histology of the liver of zebrafish after 14 days of exposure. Light micrographs of sections through the liver of zebrafish showing the histological structure of the control group **(A)** and animals treated with 1 **(B)**, 10 **(C)**, and 50 μg/L **(D)** 4,4′-di-CDE, respectively. Samples were stained with hematoxylin and eosin, and photomicrographs were taken using ×100 magnification. N, nucleus necrosis; HV, hepatocyte vacuolation; NE, nuclear enlargement.

**FIGURE 7:**
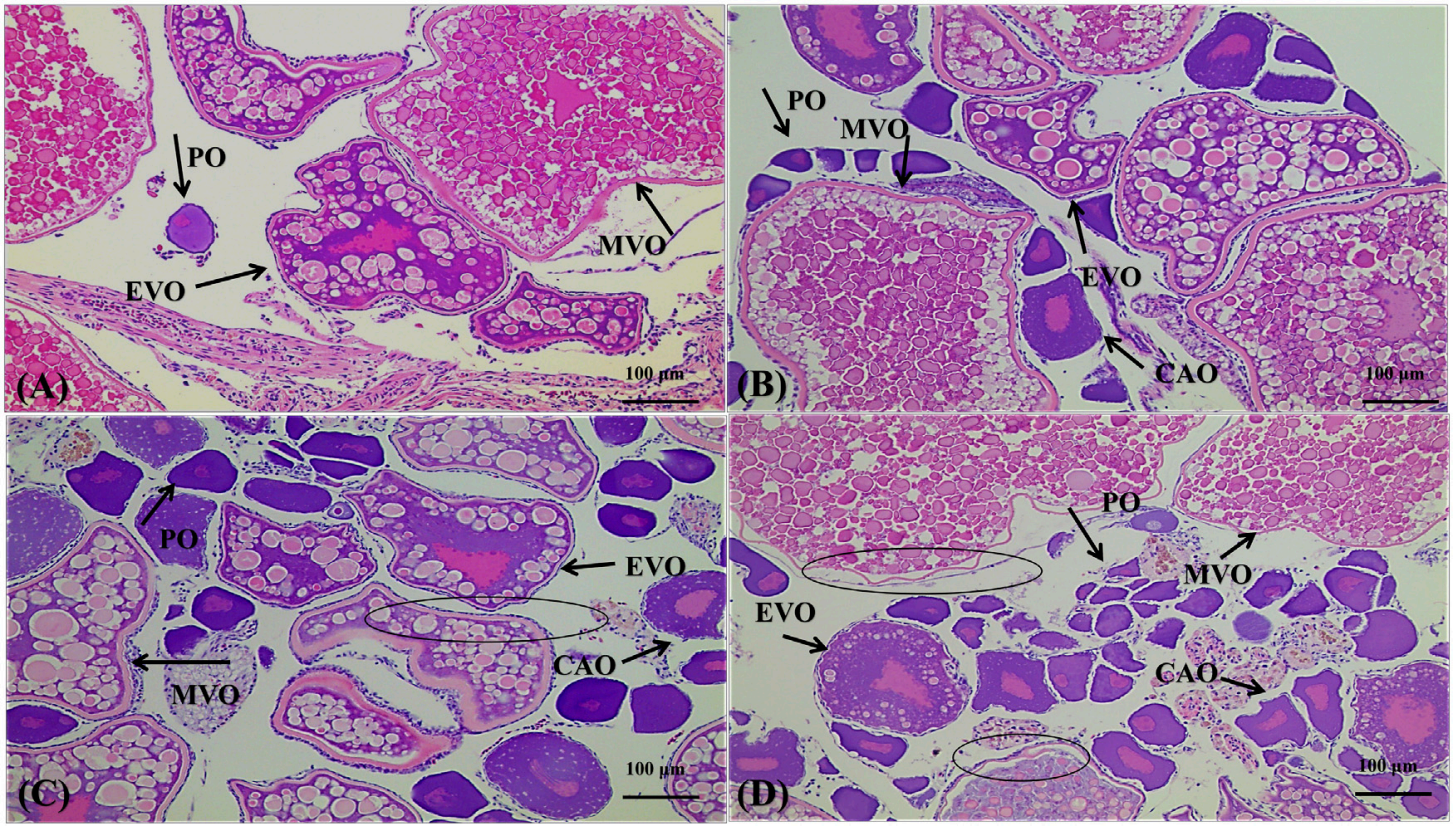
Effects of 4,4′-di-CDE exposure on histology of the ovary of zebrafish after 14 days of exposure. **(A)** Ovary from the control, showing normal ovarian structure with developmental stages of the oocytes. **(B)** Ovary from 1 μg/L group, exhibiting the number of PO and CAO increase; **(C)** and **(D)** ovary from 10 to 50 μg/L groups not only shows the number of PO and CAO increasing but also the loss of contacts between the oocyte cell membranes (ellipse). PO, peri-nuclear oocyte; CAO, cortical alveolar oocyte; EVO, early vitellogenic oocyte; MVO, mid-late vitellogenic oocyte.

In fish, the liver is the basic organ for the metabolism of toxic substances, which is vulnerable to the attack of harmful substances (Hook et al., 2018; Rodd et al., 2017; Mai et al., 2019). The attacked liver is prone to damage such as liver sinusoids, liver necrosis, hepatocyte vacuolization, and nuclear enlargement, which is closely related to liver inflammation (Bentivegna et al., 2015; Kerambrun et al., 2012). To investigate the toxicity of PCDEs in a better way, using 4,4′-di-CDE, for example, the histological photomicrographs of male liver sections and female ovary from adult zebrafish specimens are shown in Figures 6, 7. The pathological changes in the liver were different in the three concentration exposure groups. The liver sections in the control group were in a normal state in which hepatocytes and nuclei were uniform in size and shape (Figure 6A). In the 1 μg/L 4,4′-di-CDE group, zebrafish liver began to appear with histological lesions such as cell nuclei necrosis and hepatocyte vacuolation. Meanwhile, the proportion of cell nuclei necrosis and hepatocyte vacuolation increased in the 10 μg/L dose group (Figure 6B), which suggested the interference in the lipid metabolism, inhibition of protein synthesis, and energy depletion of fish (Macêdo et al., 2020). Similar results have also been noted in zebrafish exposed to tebuconazole and difenoconazole (Jiang et al., 2021). Furthermore, for the 50 μg/L dose group, the areas of nuclear necrosis and vacuolization of hepatocytes were increased and displayed new injuries, namely nuclear enlargement after exposure (Figures 6C, D). The occurrence of necrotic areas in the liver was more severe in the case of fish treated with a higher concentration group, and the probability of nuclear necrosis and enlargement increased with the increased concentration of 4,4′-di-CDE, which reflects the failure of cellular protective mechanisms in the presence of chemical stress (Olufayo and Alade, 2012). Under this case, the degree of liver inflammation may gradually increase with enhancing doses of 4,4′-di-CDE. Related research has also proved that the degree of liver damage would become more serious with an increase in exposure doses (Zhou et al., 2011).

Oxidative stress reaction can be induced by 4,4′-di-CDE in zebrafish, which can produce excessive ROS and thus results in the unsaturated fatty acids on the liver cell with membrane surface degraded to produce lipid peroxide (LPO). Eventually, hepatocytes and their organelles would be damaged to varying degrees in different groups.

In the ovary of control fish, the follicular cells at different stages were observed including the peri-nuclear oocyte (PO), cortical alveolar oocytes (CAO), early vitellogenic oocytes (EVO), and mid-late vitellogenic oocytes (MVO) (Figure 7A). No significant damage was shown in the low-dose group (Figure 7B). In the higher concentration groups, the loss of contact between the oocyte cell membranes and the follicular cell layer was found (Figures 7C, D). As seen in the graph, the number of follicle cells in the early stages increased significantly with the increase of concentration, and the proportion of mid-late vitellogenesis/mature oocytes decreased, suggesting that high doses of 4,4′-di-CDE may inhibit the development of follicle cells. Estrogen and progestin, the representative substances of endocrine, have been shown to affect oocyte development and maturation in fish (Silva et al., 2012). Exogenous endocrine disruptors with estrogen or progesterone can also have such effect. For example, ovary maturation in zebrafish was delayed when exposed to norethindrone at 84 ng/L for 90 days (Liang et al., 2020). Juvenile zebrafish exposure to norethindrone at 4.0 ng/L for 45 days resulted in increased percentages of the advanced stage of primary oocytes (Hou et al., 2018). Likewise, the observed inhibition of 4,4′-di-CDE on ovarian development indicated that it may have an estrogen interference effect.

**FIGURE 8:**
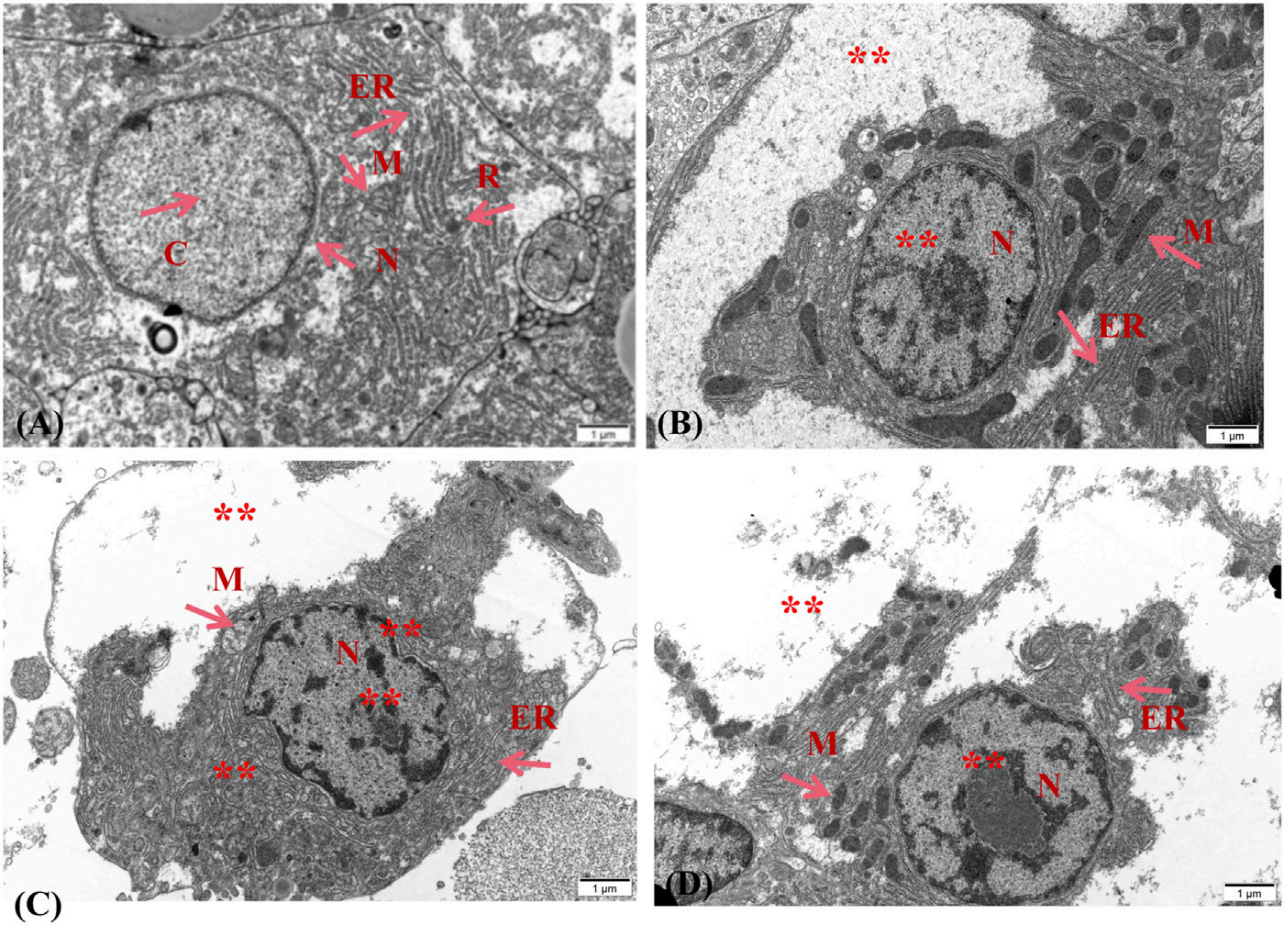
Effect of 4,4′-di-CDE on the ultrastructure of zebrafish liver after 14 days of exposure. The ultrastructure of zebrafish liver exposed to **(A)** control group, **(B)** 1 μg/L, **(C)** 10 μg/L, and **(D)** 50 μg/L 4,4′-di-CDE, and photomicrographs were taken using ×15000 magnification. N, nucleus; C, chromatin; M, mitochondria; ER, endoplasmic reticulum; R, ribosome; **, large area damage.

**FIGURE 9:**
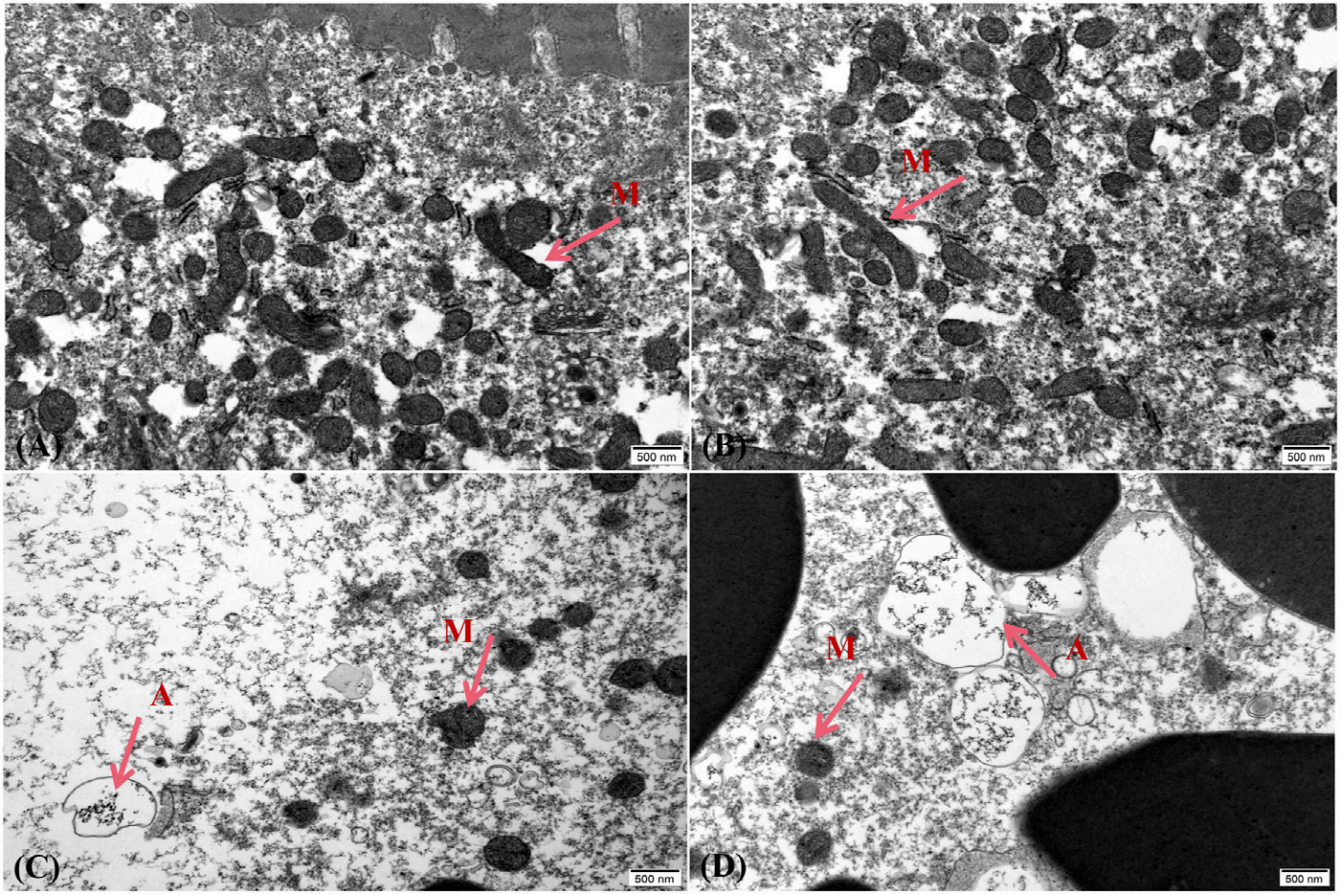
Effect of 4,4′-di-CDE on the ultrastructure of zebrafish ovary after 14 days of exposure. The ultrastructure of zebrafish ovary exposed to **(A)** control group, **(B)** 1 μg/L, **(C)** 10 μg/L, and **(D)** 50 μg/L 4,4′-di-CDE, and photomicrographs were taken using ×25000 magnification. M, mitochondria; A, autophagosome.

For further morphological study, the ultrastructure of the liver and ovary that was exposed to 4,4′-di-CDE for 14 days were analyzed and showed damage to intracellular organelles. In the control group, the structure of hepatocytes was complete and there was no pathological change in organelles (Figure 8A). The liver cells exposed to 1 and 10 μg/L showed that ribosomes were missing. The endoplasmic reticulum was broken and chromatin condensation occurred (Figures 8B, C). With the increase in exposure concentration, the damage became more and more serious. The high-dose exposure group (100 μg/L) showed liver cell rupture and loss of various organelles (Figure 8D). In the control group, the mitochondria in the ovarian cells were complete and considerable, while the treatment group (10 and 100 μg/L) showed that the number of mitochondria disintegrated and reduced with the increase in exposure concentration (Figures 9A–D). Similar results were also reflected in other studies that the endoplasmic reticulum and mitochondria in zebrafish hepatocytes were seriously damaged after exposure to higher concentrations of pollution (Yang et al., 2018; Wang et al., 2019).

### 3.4 VTG Levels

**FIGURE 10:**
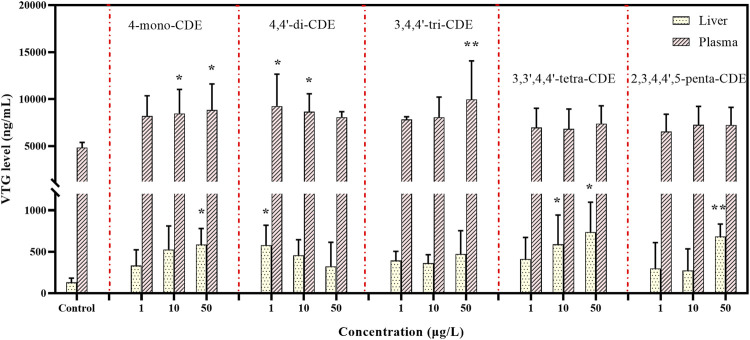
Effect of PCDEs on VTG levels in liver homogenate and plasma of male zebrafish after 14 days of exposure.

In order to verify whether PCDEs own estrogen effects, VTG contents were determined in this work. The content of VTG in juvenile and male zebrafish is small and can be negligible, but it will be induced to express under the action of exogenous estrogen (Lin and Janz, 2006; Xu et al., 2008). It would act on the endocrine system through blood circulation, which may cause corresponding variations in estrogen levels. Therefore, VTG level in male zebrafish can be used as the biomarker for investigating endocrine-disrupting chemicals. In our research (Figure 10), compared with the control groups, concentrations of VTG in liver samples increased in the PCDEs-treated groups, and a significant difference was observed in the 4-mono-CDE (50 μg/L), 4,4′-di-CDE (1 μg/L), 3,3′,4,4′-tetra-CDE (10 and 50 μg/L), and 2,3′,4,4′,5-tetra-CDE (50 μg/L) groups (*p* < 0.05 and *p* < 0.01). Meanwhile, in the blood samples, VTG concentrations also increased significantly in the 4-mono-CDE- (10 and 50 μg/L), 4,4′-di-CDE- (1 and 10 μg/L), and 3,4,4′-tri-CDE (50 μg/L)-treated groups (*p* < 0.05 and *p* < 0.01). In male zebrafish, 4-mono-CDE (100 μg/L) and 4,4′-di-CDE (1 μg/L) exposure led to an increase in VTG levels between liver and blood samples, but this phenomenon did not occur in other groups. This suggests that 4-mono-CDE and 4,4′-di-CDE have stronger estrogen-like effects than other experimental groups. In addition, for 3,3′,4,4′-tetra-CDE and 2,3′,4,4′,5-tetra-CDE treatment groups, a significant increase in VTG content was found in the liver of male zebrafish, but no obvious change was detected in the plasma, which indicates that it may take more time to induce VTG production for PCDEs with more chlorine atoms. Therefore, for more chlorinated PCDE groups, obvious changes in VTG content may be detected in plasma samples by prolonging exposure time or increasing exposure concentration.

### 3.5 mRNA Expression Levels of Related Genes

**FIGURE 11:**
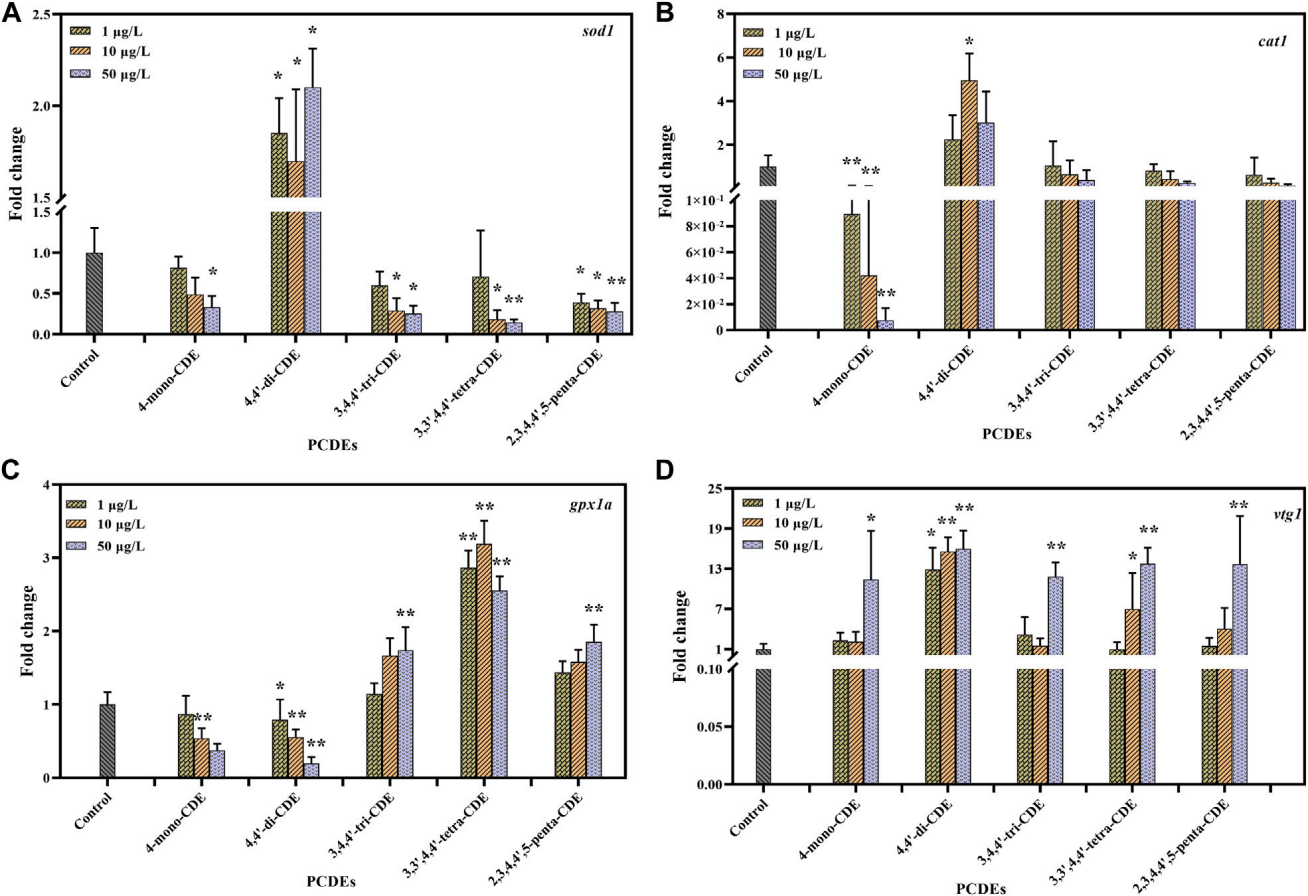
Effects of PCDEs on the mRNA expression levels of *cat1* **(A)**, *sod1* **(B)**, *gpxla* **(C)**, and *vtg1* **(D)** in the liver of adult male zebrafish. The data are presented as mean ± SD.

After the enzyme activity experiment (on the 14^th^ day), we explored the expression of genes involved in oxidative stress. Gradual downregulation of *sod1*, *cat1*, and *gpx1a* (Figure 11) expression was observed in groups exposed to 1, 10, and 50 ug/L 4-mono-CDE when compared with the control. These genes were significantly inhibited by 4-mono-CDE, indicating that the normal transcriptions were strongly inhibited as the exposure period extended. This result is in line with the findings of Li et al. (2021) who observed similar changes in zebrafish treated with 2-amino-3-methylimidazole [4,5-f] quinoline (IQ). The transcriptional levels of *sod1* and *cat1* (10 μg/L) in the zebrafish were significantly increased, while *gpx1a* was significantly inhibited in the 4,4′-di-CDE exposure groups. This phenomenon indicated that the antioxidant defense system in the liver may be mobilized to resist oxidative damage and the second line of the antioxidant defense system may have been damaged. In the medium and high dose of 3,4,4′-tri-CDE-, 3,3′,4,4′-tetra-CDE-, and 2,3′,4,4′,5-penta-CDE-treated group (Figures 11A, B), the mRNA level of *sod1* was significantly inhibited, while *cat1* have no change and *gpx1a* was significantly higher than that of the control group. This showed medium and high doses of these substances may damage the second line of the antioxidant system in the liver, forcing the significant expression of the *gpx1a* gene to eliminate excess ROS. We observed that the expression of antioxidant genes was basically consistent with the activities of SOD, CAT, and GPx. But the expression trend of related genes was different for each PCDE. We speculated that the reason may be related to the properties and structure of PCDEs.

The *gpx1a* was significantly inhibited in the lower chlorinated PCDEs (4-mono-CDE and 4,4′-di-CDE), suggesting that lower chlorinated PCDEs may force zebrafish liver to produce excessive ROS and destroy the second antioxidant defense line (Figure 11C). This indicated that the ROS produced by mid-chlorinated PCDEs (3,4,4′-tri-CDE, 3,3′,4,4′-tetra-CDE, and 2,3′,4,4′,5-penta-CDE) may have destroyed the first antioxidant defense line, but do not damage the second line of defense which was struggling to eliminate harmful substances. The log*K*_ow_ value of mid-chlorinated PCDEs is relatively higher, which means that their corresponding steric hindrance is large (Burreau et al., 1997; Boon et al., 2002; Haraguchi et al., 2009). This property can hinder their absorption and metabolism by organisms, resulting to lower ROS produced by the liver than that of low chlorinated PCDEs. Relevant studies have revealed that the ortho substitution of the chlorine atom in the ether bond of PCDE homology (i.e., Cl substitution at 2-, 2′-, 6-, and 6′- position) may lead to the distortion between the two benzene rings, thus weakening their coplanarity and reducing the toxicity of PCDEs (Personne and Janssen, 1994; Chen et al., 2007; Zhang et al., 2018). In our study, except for 4-mono-CDE, all PCDEs have ortho substitution. Therefore, this may be one of the reasons why the liver damage of low-chlorinated PCDEs is greater than that of high-chlorinated PCDEs.

In addition, in the male liver, except for 4,4′-di-CDE, the transcription levels of *vtg1* were significantly upregulated in other medium and high concentration groups of PCDEs, which suggests that PCDEs were an estrogen endocrine disruptor (Figure 11D). *Vtg1* gene was significantly upregulated in 1, 10, and 50 μg/L 4,4′-di-CDE and increased with the concentration. Other exposure groups had a similar situation where *vtg1* expression increased with dose, which suggested that a higher concentration of PCDEs promotes the expression of the *vtg1* gene. Some researchers have similar results to us (Van den Belt et al., 2003; Jin et al., 2009; Yamaguchi et al., 2015).

## 4 Conclusion

In summary, dose-dependent changes in the antioxidant enzyme activities, MDA contents, and VTG levels in the liver of zebrafish were observed after *in vivo* PCDE exposure, and the transcriptional levels of genes (*sod1*, *cat1*, and *gpx1a*) that are related to antioxidant biomarkers and *vtg1* were also significantly influenced. Higher toxicity 4,4′-di-CDE exposure caused severe liver and ovary tissue damage after 14 days of exposure. Among five PCDE congeners, 4-mono-CDE and 4,4′-di-CDE induced more severe oxidative stress in zebrafish tissues. Both histological examinations and TEM observation provided evidence for tissue and cell injuries after that resulting from 4,4′-di-CDE exposure. Five tested PCDE congeners also significantly altered VTG content and related gene *vtg1*, suggesting their potential estrogen disrupting effects. These findings provide useful information on the understanding of the toxic effects of PCDEs in fish, as well as enriched toxicological data of PCDEs on aquatic species and contribute to evaluating the threats of PCDEs to the aquatic environment. Furthermore, studies to elucidate the specific mechanisms on the endocrine disruptive effects and developmental toxicity of PCDEs in fish are warranted in the future, such as endocrine disruptive effects, which are our next research goals.

## Data Availability

The original contributions presented in the study are included in the article/Supplementary Material; further inquiries can be directed to the corresponding authors.
