# Corrigendum: Biomarker responses, gene expression alterations, and histological changes in zebrafish (*Danio rerio*) after *in vivo* exposure to polychlorinated diphenyl ethers

**DOI:** 10.3389/fphys.2022.1000714

**Published:** 2022-08-19

**Authors:** Chunmeng Ye, Wenli Xiong, Shuaishuai Shi, Jiaqi Shi, Wenhui Yang, Xuesheng Zhang

**Affiliations:** ^1^ School of Resources and Environmental Engineering, Anhui University, Hefei, China; ^2^ Laboratory of Wetland Protection and Ecological Restoration, Anhui University, Hefei, China; ^3^ Nanjing Institute of Environmental Sciences of the Ministry of Ecology and Environment, Nanjing, China

**Keywords:** polychlorinated diphenyl ethers, oxidative stress, histological changes, integrated biomarker response, endocrine disrupting effects

In the published article, there was an error in Figure 6 as published. The incorrect image for Figure 6D was used. The corrected Figure 6 appears below.

**FIGURE 6:**
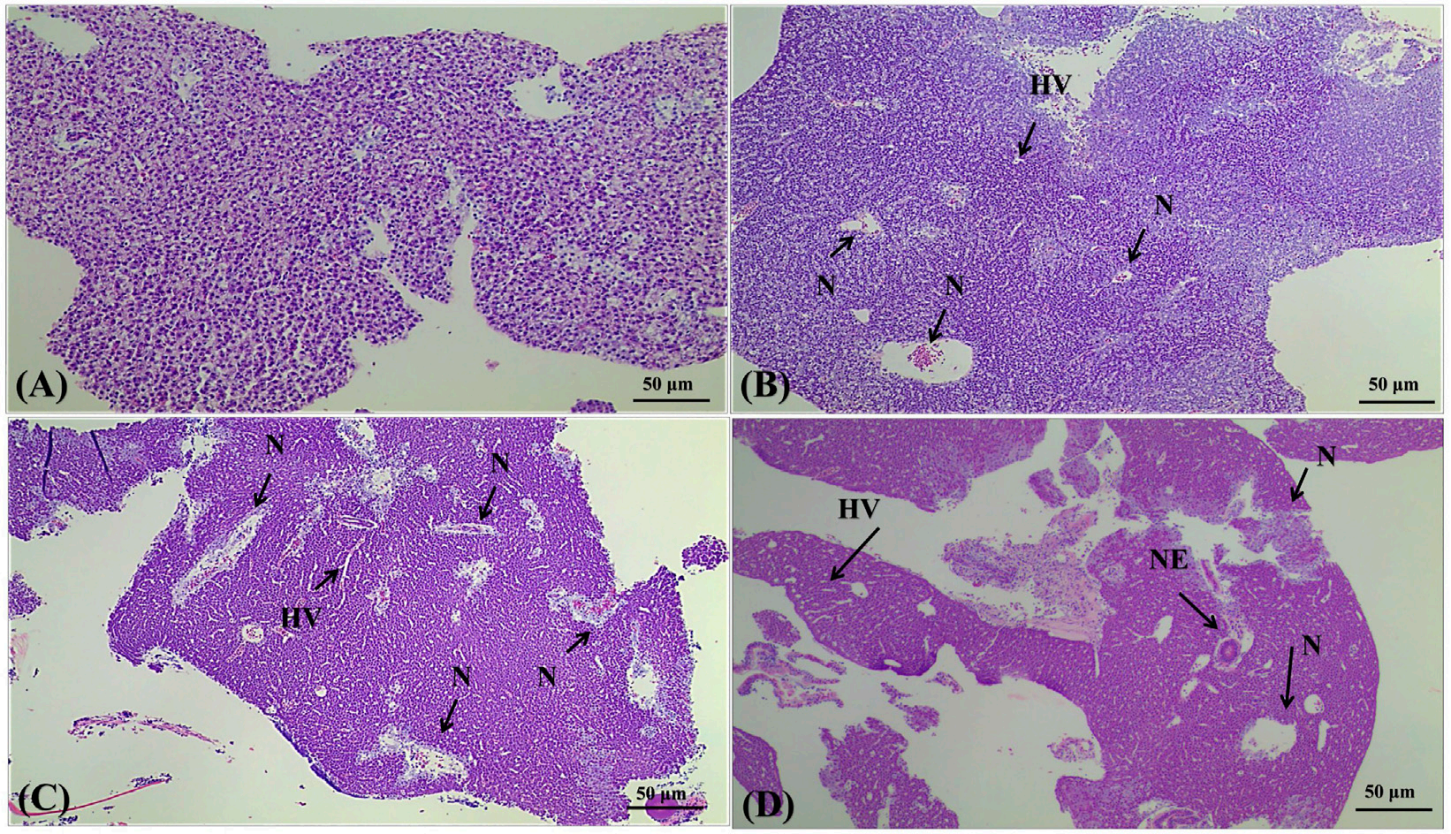
Effects of 4,4′-di-CDE exposure on histology of the liver of zebrafish after 14 days of exposure Lights micrographs of sections through the liver of zebrafish showing the histological structure od the control group**(A)** and animals treated with 1 **(B)**, 10 **(C)**, and 50 μg/L **(D)** 4,4′-di-CDE, respectively. Samples were stained with hematoxylin and eosin and photomicrographs were taken using ×100 magnification. N-Nuclei Necrosis, HV-hepatocyte vacuolation, NE-Nuclear Enlargement.

The authors apologize for this error and state that this does not change the scientific conclusions of the article in any way. The original article has been updated.

