# Supplementary material for: Biomarker Responses, Gene Expression Alterations, and Histological Changes in Zebrafish (Danio rerio) After In Vivo Exposure to Polychlorinated Diphenyl Ethers
Source: Front Physiol. 2022 Jun 3;13:907906. doi: 10.3389/fphys.2022.907906 (PMC9203962; doi:10.3389/fphys.2022.907906)
Supplement: Supplementary file 1 [file DataSheet1.PDF]

Supplementary Material

Biomarker responses, gene expression alterations, and histological changes in zebrafish (*Danio rerio*) after *in vivo* exposure to polychlorinated diphenyl ethers

Chunmeng Ye[a,b], Wenli Xiong[a,b], Shuaishuai Shi[a,b], Jiaqi Shi[c,*], Wenhui Yang[a,b], Xuesheng Zhang[a,b,*]

[a] *School of Resources and Environmental Engineering, Anhui University, Anhui Hefei 230601, China*

[b] *Laboratory of Wetland Protection and Ecological Restoration, Anhui University, Anhui Hefei 230601, China*

[c] *Nanjing Institute of Environmental Sciences of the Ministry of Ecology and Environment, Jiangsu Nanjing 210042, China*

20 Pages

3 Tables

1 Figures

[*]***Authors for correspondence.***

[1] School of Resources and Environmental Engineering, Anhui University, Anhui Hefei 230601, China. E-mail address: zhangqing8725@163.com (X.-S. Zhang);

[2] Nanjing Institute of Environmental Sciences of the Ministry of Ecology and Environment, Jiangsu Nanjing 210042, China. E-mail address: shijiaqi_jiayou@163.com (J.-Q. Shi).

Chemicals and Materials

Tissue fixative (Composition: 4% paraformaldehyde in PBS) was purchased from Dalian Meilun Biotrchnology Co., Ltd and DMSO, 2.5% glutaraldehyde and other solvents of AR grade were provided by Shanghai Aladdin Biochemical Technology Co., Ltd. (Aladdin, Shanghai, China). Heparin sodium anticoagulant comes from Beijing Dingguo Changsheng Biotechnology Co., Ltd. Analytical reagent (AR) grade silica gel (100-200 mesh), glacial acetic acid, anhydrous ethanol, sodium chloride， magnesium sulfate anhydrous, and sodium sulfate anhydrous were supplied by Sinopharm Chemical Reagent Co., Ltd. (Shanghai, China). Ultrapure water used throughout the whole experiment was produced via a Millipore Purification System (Millipore Elix 20, Millipore Corporation, USA). Pesticide analysis grade methanol, n-hexane and dichloromethane were purchased from Tedia Ltd. (Ohio, USA). Materials used in accelerated solvent extraction were purchased from Dionex Company (Sunnyvale, USA). Solid-phase extraction (SPE) columns packed with C_{18} sorbent (1 g, 6 mL) were purchased from Anpel Company (Shanghai, China).

Extraction of 5 types of PCDE congeners

The test medium contained impurities, i.e., inorganic salts, which can significantly influence the determination of PCDEs. Hence, the SPE treatment was used to extract PCDEs from the test solutions (Zhang et al. 2018; Qin et al. 2015). A C_{18}-SPE column (1 g, 6 mL) was used to enrich the target compounds. In brief, before the extraction, 10-100 mL (according to the nominal concentrations) of the test solutions was filtered using a glass fiber membrane (0.45 μm). First, C_{18} SPE columns were successively washed with 6 mL *n*-hexane and dichloromethane (2:1, v/v), 6 mL methanol, and 6 mL ultrapure water. Second, the test solutions were directed through the activated SPE columns at a constant flow rate (3 mL/min) under a low vacuum. Third, the SPE columns were washed with a 6 mL mixture of methanol and water (1:1, v/v). The two target compounds were eluted with 6 mL of dichloromethane and *n*-hexane (2:1, v/v). Finally, the eluent was dried with anhydrous sodium sulfate (Na_2SO_4), reduced to 1 mL using a rotary evaporator, and then 1 mL of concentrates were dried under a gentle N_2 stream. Before injection, the volume was brought to 1 mL with *n*-hexane. The concentrations of PCDEs in the test solutions were determined using GC-MS analysis.

GC-MS Analysis

5 different kinds of PCDEs were analyzed using GC-MS (Trace DSQ II, Thermo Scientific, USA). The injector temperature was set at 250 °C, and the helium (He, purity 99.999%) was used as the carrier gas at a constant flow speed of 1.0 mL min^{-1}. 1 μL sample was injected into the GC-MS equipped with a DB-5MS capillary column (0.25 mm × 30 m, 0.25 μm, J&K Scientific, USA) at the splitless injection mode. The GC temperature-programming was optimized as follows: the initial column temperature was 60 °C and held at for 2 min, then increased at 10 °C min^{-1} to 180 °C and held for 1 min, then increased at 5 °C min^{-1} to 240 °C and held for 1 min, finally increased at 15 °C min^{-1} to 300 °C and held for 5 min. The ion source temperature was set at 240 °C and the transfer line temperature was set at 280 °C. The quadrupole mass spectrometer was operated in the electron ionization (EI) mode. Quantitative determination of PCDE isomers was performed using the selected ion-monitoring (SIM) mode (Figure S1, Table S3).

Preparation of calibration curves

Ten mg of each PCDE congener was accurately weighed, transferred to a 100 mL volumetric flask, and brought to 100 mL using *n*-hexane. Subsequently, the solution was diluted to different concentration gradients ranging from 1 ppb to 1 ppm (i.e., 1 ppb, 10 ppb, 25 ppb, 50 ppb, 0.1 ppm, 0.25 ppm, 0.5 ppm, and 1 ppm) in *n*-hexane and analyzed by GC-MS. Each concentration gradient corresponded to a peak area. Afterwards, the calibration curves were established, with the concentration as the horizontal coordinates and the peak area as the ordinate. The concentrations of 5 PCDE congeners extracted from the samples were individually calculated from their corresponding calibration curves, with all their correlation coefficients (R^2) higher than 0.996.

Determination of oxidative stress biomarkers in zebrafish

SOD activity was measured according to a modified method described by Flohé and Oyying (1984a), which is based on the measurement of the inhibition of the reduction rate of cytochrome c by the superoxide radical. Absorbance was measured with a UV-VIS spectrophotometer at 550 nm. SOD activity was expressed in U/mg protein, with one U indicating the quantity of SOD that promoted the inhibition of 50% of the reduction rate of cytochrome c of 1 mg protein in 1 mL reaction liquid. CAT activity was determined according to Goth (1991), and its key method was monitored the residual H2O2 absorbance at 405 nm. CAT activity was showed in U/mg protein, with one U representing the de-composition of 1 μmol H_2O_2 in 1 mg protein per second. Glutathione peroxidase (GPx) and activity was determined according to Flohé and Gunzler (1984b). The enzyme activity was expressed as U, with 1 U representing 1 mg protein reduced GSH/ μmol/L/min. Glutathione (GSH) content was measured according to the methodology described by Diamantino (2001), and was expressed as GSH concentration in mg per protein. In addition, lipid peroxidation (LPO) in organisms was estimated from the production of MDA. MDA content was measured according to the methodology described by Devasagayam (1986). The amount of MDA formed was measured at 532 nm after reaction of the sample homogenate with thiobarbituric acid reactive substances (TBARS), and was expressed as MDA concentration in nmol per mg protein.

Determination steps of gene expression

50 mg of liver sample was removed from the refrigerator at - 80 °C, and then 1 ml Trizol and glass beads were added for grinding (5 min). The homogenate after grinding is transferred to a new EP tube, and chloroform (V: 1/5 volume of Trizol) was added, then shaken and mixed until the solution was emulsified and milky white. Subsequently, These liquids were allowed to stand at room temperature (5 min) and centrifuged (4 °C , 5000 rpm) for 15 min. At this time, the homogenate was divided into three layers: colorless supernatant (containing RNA), white protein layer in the middle and colored lower organic phase. The supernatant was transferred to another new centrifuge tube, and an equal volume of isopropanol was added to it. The centrifuge tube is gently reversed from top to bottom and fully mixed. Then, these liquids were centrifuged at 4 °C for 10 min at 5000 rpm, and the supernatant was discarded. After precipitation at room temperature for 5 min, RNase-free water was dissolved and added. The RNA concentration of each tube was measured on a Nanodrop spectrophotometer.

Integrated Biomarker Response (IBR)

The IBR index was used to integrate all results from different biomarkers and understand general responses. According to Beliaeff and Burgeot's methods, the IBR value is calculated by summing up triangular star plot areas calculated for each two neighbouring data. Normalize the data according to the following equation:

$$Y = (X - m)/s \qquad (1)$$

Where the unit of Y is the standard value for each situation; X is the mean value for the biomarker at a given concentration; m and s are the general mean and the standard deviation of all data regarding a given biomarker.

$$S = Z + |Min| \qquad (2)$$

Where Z was calculated using Z = -Y or Z = Y, in the case of a biological effect corresponds respectively to an inhibition or a stimulation; |Min| is the absolute value for the minimum value for all calculated Y in a given biomarker at all measurements made.

$$IBR = \sum_{i=1}^{n} A_i \qquad (3)$$

$$A_i = \frac{S_i}{2} \sin\beta (S_i \cos\beta + S_{i+1} \sin\beta) \qquad (4)$$

$$\beta = \tan^{-1}\left(\frac{S_{i+1}\sin\alpha}{S_i - S_{i+1}\cos\alpha}\right) \qquad (5)$$

where S_i and S_{i+1} are two consecutive clockwise scores (radius coordinates) of a given star plot; A_i corresponds to the area the connecting two scores; n the number of biomarkers and energy reserves used for calculations; and $\alpha = \frac{2\pi}{n}$.

Table S1. Detection concentration of PCDEs in biological and abiotic environmental matrix.

Environmental matrix	Region /Country	∑PCDE concentration (n)[a]	References
**Water**			
	Yangtze Rive, China	730-1800 ng·L^{-1} (15)	Qin et al., 2015
	Chaolu Lake, China	0.351-2.02 ng·L^{-1} (15)	Zhang et al., 2018
**Sediment**			
	Kymijoki river, Finland	138-561 ng·g^{-1} d.w (50)	Koistinen et al., 1995a
	Downstream of Kymijoki River, Finland	8.8-606 ng·g^{-1} d.w (40)	Lyytikäinen et al., 2003a,b
	The Great Lakes, North America	ND-5.54 ng·g^{-1} d.w (7)	Li et al., 2018
	Lake Ontario, North America	622-1929 ng·g^{-1} d.w (45)	Villeneuve et al., 1999
	Yangtze Rive, China	1.24-3.98 ng·g^{-1} d.w (15)	Qin et al., 2015
	Chaolu Lake, China	0.279-2.47 ng·g^{-1}d.w (15)	Zhang et al., 2018
**Soil**			
	Sawmill sites, Sweden	19-6800 ng·g^{-1} d.w	Persson et al., 2007
**Atmosphere**			
	Taiwan countryside, China	0.0144 pg·m^{-3} (6)	Chao et al., 2014
	Over the pacific ocean	0.00875 pg·m^{-3} (6)	Chao et al., 2014
**Fly ash**			
	Waste incineration plant, German	93 ng·g^{-1} (79)	Kurz et al., 1995
**Stack gases**			
	Japan	205-279000 ng·Nm^{-3}	Nakao et al., 2006
	Refuse incineration plant, Taiwan, China	1.48-10.3 ng·Nm^{-3} (6)	Yang et al., 2015
**Mammal**			
Human	Helsinki, Finland	9.4-28 ng·g^{-1} l.w (50)	Koistinen et al., 1995b
Seals	Bothnian Bay and Lake Saimaa, Finland	70-466 ng·g^{-1} l.w (50)	Koistinen et al., 1995a
	Hornby and Vancouver island, Canada<br>Smith and Gertrude island	6.5-21 ng·g^{-1} l.w (25)	Ross et al., 2013
	Gulf of finland	0.15-62 ng·g^{-1} l.w (50)	Koistinen et al., 1997
**Fish**			
Cod	Arctic, Norway	2.1-21 ng·g^{-1} l.w (45/50)	Koistinen et al., 1995b

Salmon	Baltic Sea, Northern Europe	92 ng·g^{-1} l.w (50)	Sinkkonen et al., 2000
	The Great Lakes, North America	10-1983 ng·g^{-1} l.w	Niimi et al., 1994
Carp	Lake Ontario, North America	766-14005 ng·g^{-1} w.w (18)	Huestis et al., 1992
Pike	Lake Ontario, North America	1053-4958 ng·g^{-1} w.w (18)	Huestis et al., 1992
	Lake Ontario, North America	21000-47000ng·g^{-1} l.w (45)	Villeneuve et al., 1999
	Kymijoki River, Finland	784-799 ng·g^{-1} l.w (50)	Koistinen et al., 1995a
Bream	Kymijoki River, Finland	642 ng·g^{-1} l.w (26)	Koistinen et al., 1993
Pickerel	Kymijoki River, Finland	545-1650 ng·g^{-1} l.w (24)	Niimi et al., 1994
Luxilus cornutus	Lake Ontario, North America	100-2857 ng·g^{-1} l.w (45)	Villeneuve et al., 1999
Luxilus cardinalis	Lake Ontario, North America	23231-43231 ng·g^{-1} l.w (45)	Villeneuve et al., 1999
Luxilus zonistius	Lake Ontario, North America	20706-96529 ng·g^{-1} l.w (45)	Villeneuve et al., 1999
Lepomis gibbosus	Lake Ontario, North America	30417-68250 ng·g^{-1} l.w (45)	Villeneuve et al., 1999
Yellow Perch	Lake Ontario, North America	4200-130333 ng·g^{-1} l.w (45)	Villeneuve et al., 1999
Ameiurus nebulosus	Lake Ontario, North America	7538-213231 ng·g^{-1} l.w (45)	Villeneuve et al., 1999
White sucker	Lake Ontario, North America	16714-174571 ng·g^{-1} l.w (45)	Villeneuve et al., 1999
**Birds**			
Haliaeetus albicilla	Baltic Sea, Northern Europe	1027-50924 ng·g^{-1} l.w (50)	Koistinen et al., 1995c
Uria aalge	Baltic Sea, Northern Europe	233-354 ng·g^{-1} l.w (50)	Koistinen et al., 1995c
Nycticorax nycticorax	Yangtze River Delta, China	11-450 ng·g^{-1} l.w (7)	Zhou et al., 2017
Chlidonias hybrida	Yangtze River Delta, China	15-700 ng·g^{-1} l.w (7)	Zhou et al., 2017
**Invertebrate**			
Unionidae	Kymijoki river, Finland	4.7-5.7 ng·g^{-1} l.w	Koistinen et al., 1997
Mytilus edulis	Narragansett Bay, America	27.6-541 ng·g^{-1} l.w (2)	Lake et al., 1981
Plankton	Lake Ontario, North America	6600-33000 ng·g^{-1} l.w (45)	Villeneuve et al., 1999
Limnodrilus	Lake Ontario, North America	5123-34947 ng·g^{-1} l.w (45)	Villeneuve et al., 1999
	Downstream of Kymijoki River, Finland	215-1325 ng·g^{-1} l.w (40)	Lyytikäinen et al., 2003b
Chironomidae	Lake Ontario, North America	4042-29292 ng·g^{-1} l.w (45)	Villeneuve et al., 1999
	Downstream of Kymijoki River, Finland	ND-1200 ng·g^{-1} l.w (40)	Lyytikäinen et al., 2003b
Gammaridea	Lake Ontario, North America	32333-66667 ng·g^{-1} l.w (45)	Villeneuve et al., 1999

**Food**			
Cod-liver oil	Germany	49-659 $ng \cdot g^{-1}$ l.w (79)	Kurz et al., 1995
Meat products	Catalunya, Spain	1.5 $ng \cdot kg^{-1}$ w.w	Martí-Cid et al., 2008
Seafood	Catalunya, Spain	1031-7088 $ng \cdot kg^{-1}$ w.w	Domingo et al., 2006<br>Martí-Cid et al., 2008
Fruits and vegetables	Catalunya, Spain	0.4-0.8 $ng \cdot kg^{-1}$ w.w	Martí-Cid et al., 2008
Eggs	Catalunya, Spain	1.5 $ng \cdot kg^{-1}$ w.w	Martí-Cid et al., 2008
Dairy products	Catalunya, Spain	0.7-1.9 $ng \cdot kg^{-1}$ w.w	Martí-Cid et al., 2008
Grain	Catalunya, Spain	0.7-1.8 $ng \cdot kg^{-1}$ w.w	Martí-Cid et al., 2008
Edible oil	Catalunya, Spain	8.3 $ng \cdot kg^{-1}$ w.w	Martí-Cid et al., 2008
Baked Goods	Catalunya, Spain	2.1 $ng \cdot kg^{-1}$ w.w	Martí-Cid et al., 2008

a: n kinds of PCDEs monomers were detected;
ND: below detection limit.

Table S2. Sequences of primers used for Real-time PCR.

Target gene	Gene ID	Primer sequences	bp
sod1	30553	F: GGCACCGTCTATTTCAATCAAGAG<br>R: GTTAGCATTGCATCCTCGATTTCA	269
cat1	30068	F: CGAGAGCGGATACCAGAGAGA<br>R: CTGCGAAACCACGAGGATCT	205
gpx1a	352926	F: GCAACCAGTTCGGGCACCAG<br>R: GGGCGTTTTCACCATTCACTTCC	130
vtg1	559475	F: ACTGAGAGGTGTGCTGAATGC<br>R: CTTGATGAATTTTATAATGACTGGTGTACC	546

Table S3. Retention time and quantitative ions of 5 types of PCDEs.

NO.	Name	Retention Time	Ions (*m/z*)
2	4-mono-CDE	14.02	204, 141, 77
4	4,4'-di-CDE	16.66	240, 238, 175
7	3,4,4'-tri-CDE	21.63	274, 272, 202
9	3,3',4,4'-tetra-CDE	24.52	308, 306, 243
11	2,3',4,4',5-penta-CDE	26.61	344, 272, 207

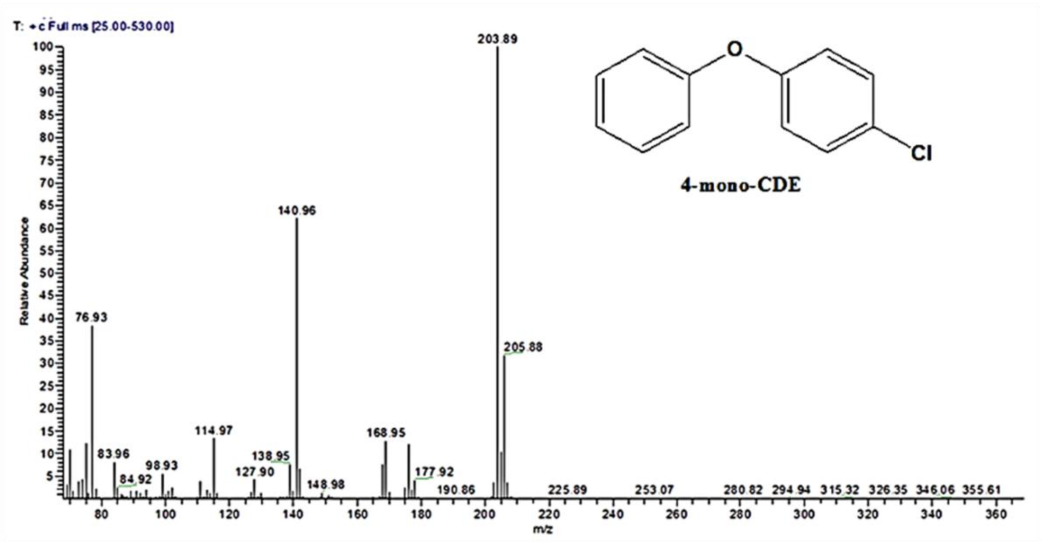
T: + c Full ms [25.00-530.00]
Relative Abundance
m/z
203.89
140.96
76.93
205.88
114.97
168.95
83.96
98.93
138.95
127.90
84.92
177.92
148.98
190.86
225.89
253.07
280.82
294.94
315.32
326.35
346.06
355.61
O
Cl
4-mono-CDE

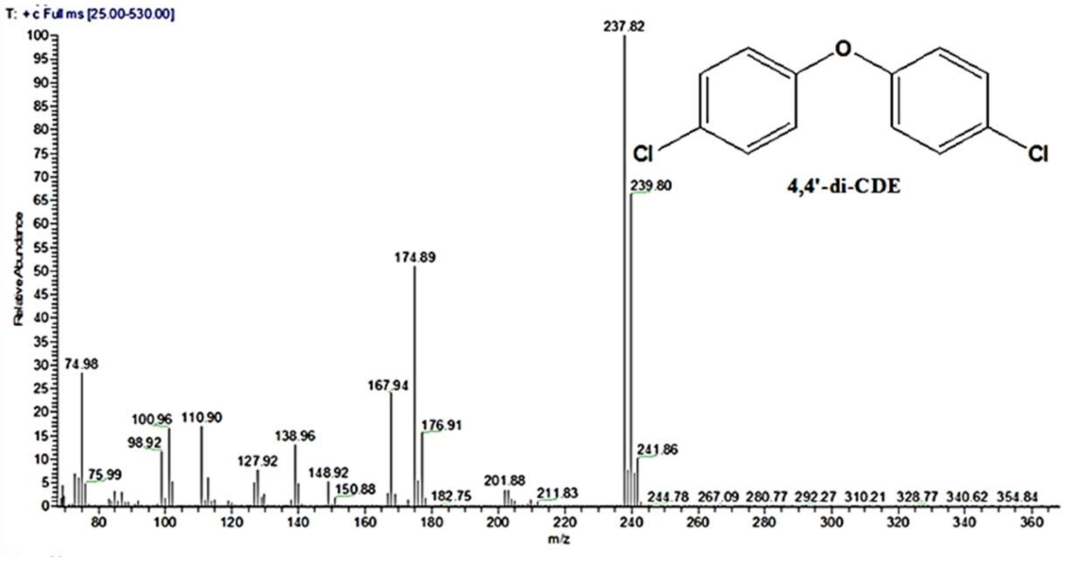
T: + c Full ms [25.00-530.00]
Relative Abundance
m/z
237.82
239.80
174.89
74.98
167.94
100.96
110.90
138.96
98.92
176.91
241.86
127.92
148.92
75.99
150.88
201.88
182.75
211.83
244.78
267.09
280.77
292.27
310.21
328.77
340.62
354.84
O
Cl
Cl
4,4'-di-CDE

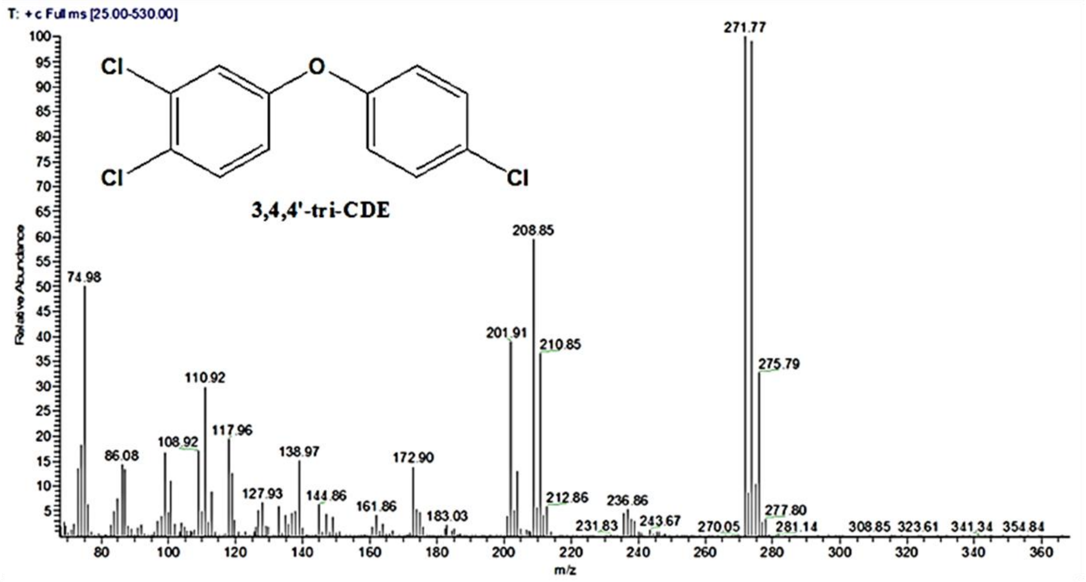
T: + c Full ms [25.00-530.00]
3,4,4'-tri-CDE
Relative Abundance
m/z
271.77
208.85
74.98
201.91
210.85
275.79
110.92
108.92
117.96
86.08
138.97
172.90
127.93
144.86
161.86
183.03
212.86
236.86
231.83
243.67
270.05
277.80
281.14
308.85
323.61
341.34
354.84

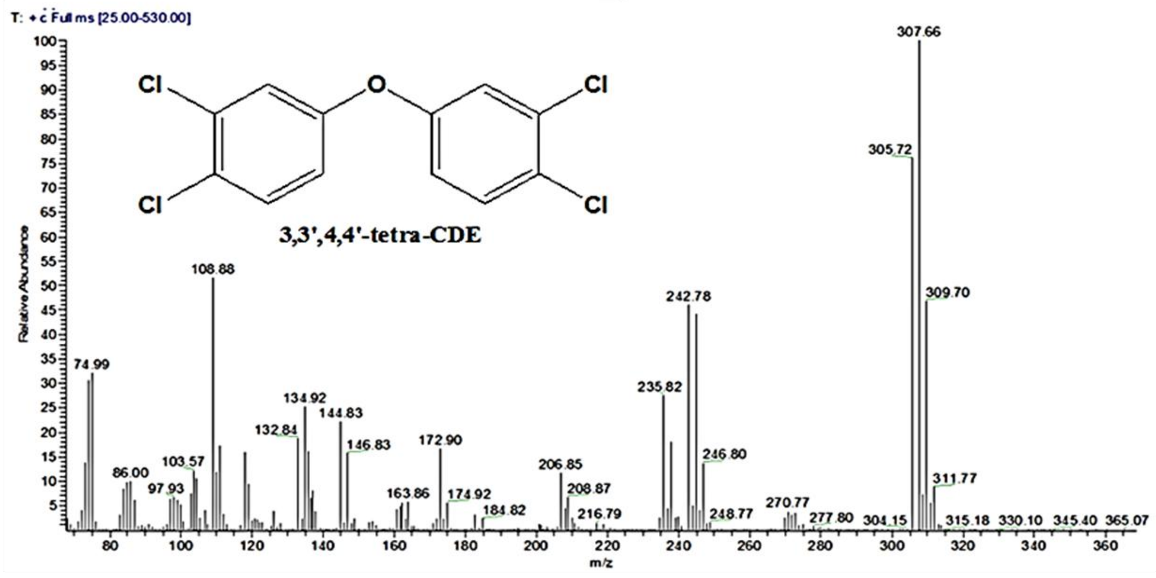
T: + c Full ms [25.00-530.00]
3,3',4,4'-tetra-CDE
Relative Abundance
m/z
307.66
305.72
108.88
242.78
309.70
74.99
235.82
134.92
144.83
132.84
146.83
172.90
86.00
103.57
97.93
163.86
174.92
206.85
208.87
246.80
311.77
184.82
216.79
248.77
270.77
277.80
304.15
315.18
330.10
345.40
365.07

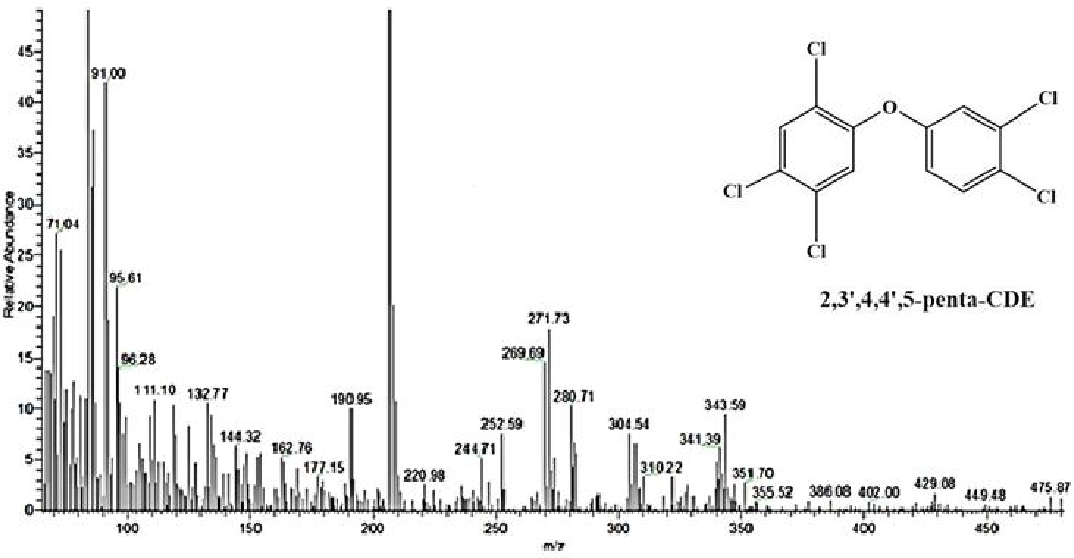

Figure S1. The GC-MS spectra of 5 types of PCDEs

REFERENCE

Zhang X.S., Wang T.T., Gao L et al. 2018. Polychlorinated diphenyl ethers (PCDEs) in surface sediments, suspended particulate matter (SPM) and surface water of Chaohu Lake, China. Environ. Pollut. 241, 441–450.

Qin L., Feng MB., Zhang X.S et al 2015. Occurrence of polychlorinated diphenyl ethers in Nanjing section of the Yangtze River: level and distribution pattern. Environ. Sci. Pollut. Res. 22, 9224–9232.

Flohé L., Oyying F., 1984a. Superoxide dismutase assays. Methods Enzymol. 105, 93–104.

Flohé L., Gunzler W.A., 1984b. Assays of glutathione peroxidase. Methods Enzymol. 105, 114–121.

Goth, L., 1991. A simple method for determination of serum catalase activity and revision of reference range. Clin. Chim. Acta., 196, 143–151.

Devasagayam T.P.A., 1986. Lipid peroxidation in rat uterus. BBA-MOL Cell Biol. L. 876, 507–514.

Diamantino T.C., Almeida E., Soares A.M., Guilhermino, L., 2001. Lactate dehydrogenase activity as an effect criterion in toxicity tests with Daphnia magna Straus. Chemosphere 45, 553–560.

Beliaeff B., Burgeot T. 2002. Integrated biomarker response: a useful tool for ecological risk assessment. Environ. Toxicol. Chem. 21, 1316–1322.

Qin L., Feng M.B., Zhang X.S et al 2015. Occurrence of polychlorinated diphenyl ethers in Nanjing section of the Yangtze River: level and distribution pattern. Environ. Sci. Pollut. Res. 22, 9224–9232.

Zhang X.S., Wang T.T., Gao L et al 2018. Polychlorinated diphenyl ethers (PCDEs) in surface

sediments, suspended particulate matter (SPM) and surface water of Chaohu Lake, China. Environ. Pollut. 2018, 241, 441–450.

Koistinen J., Paasivirta J., Suonpera M. 1995a. Contamination of pike and sediments from Kymijoki River by PCDEs, PCDDs, and PCDFs: contents and patterns compared to pike and sediment from the Bothnian Bay and seals from Lake Saimaa. Environ. Sci. Technol. 29, 2541–2547.

Lyytikäinen M., Rantalainen A.L., Mikkelson P et al. 2003a. Similarities in bioaccumulation patterns of polychlorinated dibenzop-dioxins and furans and polychlorinated diphenyl ethers in laboratory-exposed oligochaetes and semipermeable membrane devices and in field-collected chironomids. Environ. Toxicol. Chem. 22, 2405–2415.

Lyytikäinen M., Hirva P., Minkkinen P et al. 2003b. Bioavailability of sediment-associated PCDD/Fs and PCDEs: Relative importance of contaminant and sediment characteristics and biological factors . Environ. Sci. Technol. 37, 3926–3934.

Li A., Guo J., Li Z., et al. 2018. Legacy polychlorinated organic pollutants in the sediment of the Great Lakes. J. Great. Lakes. Res. 44, 682–692.

Villeneuve J.Y., Niimi A.J., Metcalfe C.D. 1999. Distribution and bioaccumulation of chlorinated diphenyl ethers in a contaminated embayment of Lake Ontario. J. Great. Lakes. Res. 25, 760–771.

Persson Y., Lundstedt S., Öberg L., et al. 2007. Levels of chlorinated compounds (CPs, PCPPs, PCDEs, PCDFs and PCDDs) in soils at contaminated sawmill sites in Sweden. Chemosphere 6, 234–242.

Chao H.R., Lin D.Y., Chen K.Y., et al. 2014. Atmospheric concentrations of persistent organic

pollutants over the Pacific Ocean near southern Taiwan and the northern Philippines. Sci. Total. Environ. 491–492, 51–59.

Kurz J., Ballschmiter K.1 995. Isomer specific determination of 79 polychlorinated diphenyl ethers (PCDE) in cod liver oils, chlorophenols and a fly ash. Fresenius J. Anal. Chem. 351, 98–109.

Nakao T., Aozasa O., Ohta S., et al. 2006. Formation of toxic chemicals including dioxin-related compounds by combustion from a small home waste incinerator. Chemosphere 62, 459–468.

Yang J.S., Lin S.L., Lin T.C et al. 2015. Emissions of polychlorinated diphenyl ethers from a municipal solid waste incinerator during the start-up operation. J. Hazard. Mater. 299: 206–214.

Koistinen J., Mussalo-Rauhamaa H., Paasivirta J. 1995b. Polychlorinated diphenyl ethers, dibenzo-p-dioxins and dibenzofurans in Finnish human tissues compared to environmental samples. Chemosphere 31, 4259–4271.

Ross P.S., Noël M., Lambourn D et al. 2013. Declining concentrations of persistent PCBs, PBDEs, PCDEs, and PCNs in harbor seals (Phoca vitulina) from the Salish Sea. Prog. Oceanogr. 115, 160–170.

Koistinen J., Stenman O., Haahti H et al. 1997. Polychlorinated diphenyl ethers, dibenzo-p-dioxins, dibenzofurans and biphenyls in seals and sediment from the Gulf of Finland. Chemosphere 35, 1249–1269.

Sinkkonen S., Paasivirta J. 2000. Polychlorinated organic compounds in the Arctic cod liver: trends and profiles. Chemosphere 40, 619–626.

Niimi A.J., Huestis S.Y., Metcalfe C.D. 1994. Chlorinated diphenyl ethers in Great Lakes fish and their environmental implication. Environ. Toxicol. Chem. 13, 1133–1138.

Huestis S.Y., Sergeant D.B. 1992. Removal of chlorinated diphenyl ether interferences for analyses of PCDDs and PCDFs in fish. Chemosphere 24, 537–545.

Koistinen J, Paasivirta J, Lathiperä M. 1993. Bioaccumulation of dioxins, coplanar PCBs, PCDEs, HxCNs, R-PCNs, R-PCPHs and R-PCBBs in fish from a pulp-mill recipient watercourse. Chemosphere 27, 149–156.

Koistinen J., Koivusaari J., Nuuja I et al. 1995c. PCDEs, PCBs, PCDDs and PCDFs in black guillemots and white-tailed sea eagles from the Baltic Sea. Chemosphere 30, 1671–1684.

Zhou Y., Yin G., Asplund L.et al. 2017. Bergman Å. Human exposure to PCDDs and their precursors from heron and tern eggs in the Yangtze River Delta indicate PCP origin. Environ. Pollut. 225, 184–192.

Koistinen J., Herve S., Paukku R. et al. 1997. Chloroaromatic pollutants in mussels incubated in two finnish watercourses polluted by industry. Chemosphere 34, 2553–2569.

Lake J.L., Rogerson P.F., Norwood C.B.A. 1981. polychlorinated dibenzofuran and related compounds in an estuarine system. Environ. Sci. Technol. 15, 549–553.

Martí-Cid R., Llobet J.M., Castell V. et al. 2008. Human exposure to polychlorinated naphthalenes and polychlorinated diphenyl ethers from foods in Catalonia, Spain: Temporal trend. Environ. Sci. Technol. 42, 4195–4201.

Domingo J.L., Bocio A., Falcó G., et al. Exposure to PBDEs and PCDEs associated with the consumption of edible marine species. Environ. Sci. Technol. 40, 4394–4399.
